# Association between novel genetic variants of Notch signaling pathway genes and survival of hepatitis B virus‐related hepatocellular carcinoma

**DOI:** 10.1002/cam4.7040

**Published:** 2024-04-01

**Authors:** Liming Qin, Moqin Qiu, Qiuling Lin, Binbin Jiang, Shicheng Zhan, Xueyan Wei, Junjie Wei, Yingchun Liu, Qiuping Wen, Peiqin Chen, Yanji Jiang, Zihan Zhou, Xiumei Liang, Ji Cao, Yizhen Gong, Yuying Wei, Xiaoxia Wei, Hongping Yu

**Affiliations:** ^1^ Department of Experimental Research Guangxi Medical University Cancer Hospital Nanning China; ^2^ Department of Epidemiology and Health Statistics, School of Public Health Guangxi Medical University Nanning China; ^3^ Department of Respiratory Oncology Guangxi Medical University Cancer Hospital Nanning China; ^4^ Department of Clinical Research Guangxi Medical University Cancer Hospital Nanning China; ^5^ Department of Scientific Research Guangxi Medical University Cancer Hospital Nanning China; ^6^ Department of Cancer Prevention and Control Guangxi Medical University Cancer Hospital Nanning China; ^7^ Department of Disease Process Management Guangxi Medical University Cancer Hospital Nanning China; ^8^ Key Laboratory of Early Prevention and Treatment for Regional High Frequency Tumor (Guangxi Medical University), Ministry of Education Nanning China; ^9^ Key Cultivated Laboratory of Cancer Molecular Medicine of Guangxi Health Commission Guangxi Medical University Cancer Hospital Nanning China

**Keywords:** hepatitis B virus, hepatocellular carcinoma, Notch signaling pathway, single nucleotide polymorphism, survival

## Abstract

**Background:**

Although the Notch pathway plays an important role in formation and progression of hepatocellular carcinoma (HCC), few studies have reported the associations between functional genetic variants and the survival of hepatitis B virus (HBV)‐related HCC.

**Methods:**

In the present study, we performed multivariable Cox proportional hazard regression analysis to evaluate associations between 36,101 SNPs in 264 Notch pathway‐related genes and overall survival (OS) of 866 patients with HBV‐related HCC.

**Results:**

It was found that three independent SNPs (*NEURL1B* rs4868192, *CNTN1* rs444927 and *FCER2* rs1990975) were significantly associated with the HBV‐related HCC OS. The number of protective genotypes (NPGs) were significantly associated with better survival in a dose‐response manner (*p*_trend_ <0.001). Compared with the model with sole clinical factors, the addition of protective genotypes to the predict models significantly increased the AUC, i.e., from 72.72% to 75.13% (*p* = 0.002) and from 72.04% to 74.76 (*p* = 0.004) for 3‐year and 5‐year OS, respectively. The expression quantitative trait loci (eQTL) analysis further revealed that the rs4868192 C allele was associated with lower mRNA expression levels of *NEURL1B* in the whole blood (*p* = 1.71 × 10^‐3^), while the rs1990975 T allele was correlated with higher mRNA expression levels of *FCER2* in the whole blood and normal liver tissues (*p* = 3.51 × 10^−5^ and 0.033, respectively).

**Conclusions:**

Three potentially functional SNPs of *NEURL1B*, *CNTN1* and *FCER2* may serve as potential prognostic biomarkers for HBV‐related HCC.

## Graphical abstract

In this study, we adopted a two‐stage study design to explore the associations between single nucleotide polymorphisms (SNPs) in the Notch pathway genes and the survival of HBV‐related hepatocellular carcinoma (HCC) patients. We found three independent SNPs (NEURL1B rs4868192 T>C, CNTN1 rs444927 G>A, and FCER2 rs1990975 C>T) were significantly associated with the OS of HBV‐related HCC, which may serve as potential prognostic biomarkers. Our study could provide reference for improving the survival of HCC.

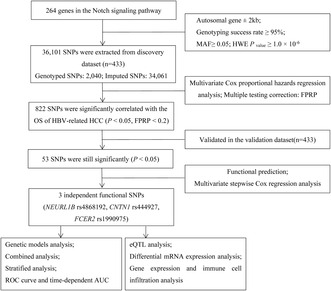

## 1 INTRODUCTION

Primary liver cancer is one of the most common malignant tumors in the world. According to the global cancer statistics, liver cancer is the sixth most common malignant tumor and the third leading cause of cancer death worldwide in 2020.^1^ Hepatocellular carcinoma (HCC) is the most important tissue type, accounting for about 75%–85% of the primary liver cancer. Previously established environmental risk factors for HCC include hepatitis C virus (HCV), alcohol abuse, alcoholic and nonalcoholic fatty liver disease, diabetes, obesity, and aflatoxin exposure. Most cases are attributed to chronic hepatitis B virus (HBV) infection in China.^2^ Due to the characteristics of occult onset, high malignancy, easy recurrence, and metastasis, the 5‐year survival rate is still poor with only 18%, even though the treatments become more available and advanced.^3^, ^4^ Therefore, it becomes urgent to identify novel effective biomarkers for improving the early survival rate of HCC.

As the most common form of human heritable variation in determining individual differences, single nucleotide polymorphisms (SNPs) may affect the expression of corresponding genes or protein translation, resulting in different susceptibility and prognosis of individuals.^5^, ^6^ Genome‐wide association study (GWAS) has found a number of susceptibility loci associated with the onset and prognosis of human complex diseases such as cancers, which provide important clues for further understanding genetic mechanisms of these complex diseases. However, GWAS may miss some important SNPs that do not reach the strictly significant level of genome‐wide association (*p* < 1.0 × 10^−7^), and the functions of most SNPs located in the noncoding region of the gene remains unclear.^7^, ^8^ In the post‐GWAS era, the method based on the biological pathway is a hypothesis‐driven method, which can use the existing GWAS dataset to identify functional reasonable train/disease‐related SNPs, and further help understand the susceptibility and prognosis mechanisms of complex diseases.^9^

The Notch pathway is a highly conserved signaling pathway that plays an significant role in cell proliferation, differentiation, apoptosis, adhesion, epithelial–mesenchymal transformation (EMT) and tumor angiogenesis.^10^ Notch signaling pathway consists of four Notch receptors (Notch 1–4) and five Notch ligands (JAG1, JAG2, DLL1, DLL3, and DLL4).^11^ The ligand–receptor interaction is the initiation of the Notch signaling pathway, while its abnormal activation or structural activation is closely related to a variety of malignant tumors.^12^ Studies showed that the mutations and disorders of Notch signaling pathway genes have dual biological functions in tumor occurrence and progression, acting as an oncogene or a tumor suppressor, depending on the cellular and microenvironment.^13^, ^14^, ^15^ In HCC, numerous studies have emphasized the carcinogenic effect of this pathway.^16^ The high expression of JAG1 in HCC was associated with HCC patients with earlier onset and lower albumin levels,^17^ and the expression and activation of Notch1‐4 were positively associated with the invasion, differentiation, and angiogenesis of HCC.^18^, ^19^ These studies suggest that the Notch signaling pathway may play an important role in HCC.

At present, few studies had investigated the associations between SNPs in the Notch pathway‐related genes and survival of HBV‐related HCC patients, though the pathway may play an important role in HCC. It is therefore hypothesized here that the genetic variants in Notch signaling pathway‐related genes are associated with survival of HBV‐related HCC patients. In this study, we adopted a two‐stage study design to test the hypothesis in 866 patients with HBV‐related HCC.

## 2 MATERIALS AND METHODS

### 2.1 Studied population

The population under study consisted of 866 patients who were first diagnosed and underwent hepatectomy at the Guangxi Medical University Cancer Hospital from July 2007 to December 2017.^20^, ^21^ All patients were confirmed as HBV‐related HCC patients by pathological examination and HbsAg seropositivity and underwent hepatectomy. Those patients who received preoperative antitumor treatments such as transcatheter arterial chemoembolization and radiofrequency ablation were excluded. The specific inclusion and exclusion criteria have been described in previous studies.^20^, ^21^ The demographic data and clinicopathological characteristics of patients, including age, sex, smoking status, drinking status, alpha‐fetoprotein (AFP) level, cirrhosis, cancer embolus, and Barcelona clinic liver cancer (BCLC) stage, were collected by consulting the patient's medical records and telephone follow‐up. All patients were followed up until death or March 31, 2020. In this study, the overall survival (OS) was defined as the time from the time of hepatectomy to death or the last effective follow‐up. The 866 patients were randomly divided into two groups, that is, discovery group and validation group, according to a 1:1 ratio. All patients have signed informed consent forms, and this study was conducted with the approval of the Institutional Review Committee of the Guangxi Medical University Cancer Hospital (LW2023052).

### 2.2 Genotyping, quality control, and imputation

Genotyping was performed with the Illumina GSA genotyping chip (Infinium Global Screening Array‐24 v1.0).^20^ PLINK1.90 software was used for systematic quality control. Based on the International 1000 Genome Sequencing Program (1000G, Phase III), Minimac3 was used for imputation (https://imputationserver.sph.umich.edu/index.htm). The inclusion criteria for imputation included (1) info score ≥0.3, (2) genotyping success rate ≥ 95%, (3) minor allele frequency (MAF) ≥ 1%, and (4) Hardy–Weinberg equilibrium (HWE) *p* < 1.0 × 10^−6^.

### 2.3 SNP selection of Notch signaling pathway genes

The genes involved in the Notch signaling pathway were selected by the Molecular Signatures Database (http://www.gsea‐msigdb.org/gsea/login.jsp) and the PathCards Database (https://pathcards.genecards.org/) with the keyword “Notch.” According to the human genome (hg19) reference, after removing duplicate genes and genes on the X chromosome, 264 genes located on autosome were left as candidate genes for further analysis (Table S1). The screening criteria for SNPs are (1) SNPs in these genes and within their ±2 kb flanking regions, (2) MAF ≥0.05, (3) the success rate of genotyping ≥95%, and (4) HWE *p* ≥ 1.0 × 10^−6^. Finally, a total of 36,100 SNPs were available for further analysis.

### 2.4 Statistical analysis

We used multivariate Cox proportional hazard regression analysis to analyze associations between the SNPs of Notch signaling pathway‐related genes and the OS of HBV‐related HCC in the additive genetic model using the “GenABEL” and “gap” package of R software (version 3.1.3), adjusted for age, sex, smoking status, drinking status, AFP level, cirrhosis, cancer embolus, and BCLC stage. We also calculated the 95% confidence interval (CI) for the hazard ratio (HR). A false positive report rate (FPRP) with a cutoff value of 0.2 was used for multiple testing correction to reduce the potential of false positive results. Furthermore, we investigated the associations between the combined protective genotypes of significant SNPs and OS of the HBV‐related HCC by each clinical variables, and the possible interaction between the combined protective genotypes and each clinical variables on the HBV‐HCC survival. Kaplan–Meier plot was used to draw the survival curve to visualize the survival difference among genotypes of SNPs, and the log‐rank test was used to compare the survival rate. The “timeROC” package of R software (version 3.5.2) was used to depict the receiver operator characteristic (ROC) curve and the area under the curve (AUC) to further investigate the predictive accuracy of the model with clinical variables and combined genotypes. ROC is a curve drawn by combining sensitivity and specificity, which measures the accuracy of model prediction by calculating AUC. All statistical analyses were performed by R software, and all tests were two‐sided, if *p* < 0.05, the difference was considered statistically significant.

### 2.5 Function prediction

SNP Info (https://manticore.niehs.nih.gov/snpinfo/snpfunc.html), Regulome DB (https://www.regulomedb.org/regulome‐search/), and HaploRegv4.1 (https://pubs.broadinstitute.org/mammals/haploreg/haploreg.php) were used to predict the function of significant SNPs. The Genotype Tissue Expression (GTEx) database (https://www.gtexportal.org/home/testyourown) was adopted to perform the expression quantitative trait loci (eQTL) to evaluate the associations between SNPs and corresponding genes mRNA expression levels. The *p*‐value of eQTL analysis was calculated by using a linear regression model. In addition, we also compared the target gene mRNA expression between normal tissues and HCC issues via TNMplot database (https://tnmplot.com/analysis/), and compared the survival difference in the mRNA expression levels of genes via Kaplan–Meier Plotter (https://kmplot.com/analysis/). Tumor IMmune Estimation Resource (TIMER2.0, http://timer.cistrome.org/) was applied to analyze the associations between the expression levels of genes and the immune cell infiltration levels in the tumor microenvironment.

## 3 RESULTS

### 3.1 Characteristics of patients and analysis of HCC OS

The clinicopathological characteristics of all patients have been described in previous studies.^20^ A total of 866 HBV‐related HCC patients were recruited, with a median follow‐up time of 39.05 months and a median age of 47 years. Multivariate analysis showed that the OS of older patients (>47 years) (HR = 0.81, 95% CI = 0.66–0.99) is better than that of younger patients (≤47 years). In addition, the patients with high AFP level (HR = 1.29, 95% CI = 1.05–1.57) or with cancer embolus (HR = 1.74, 95% CI = 1.38–2.21) and BCLC B/C stage (HR = 1.98, 95% CI = 1.56–2.52) have a worse OS, compared with those with low AFP level or without cancer embolus and BCLC 0/A stage.

### 3.2 Associations between SNPs of Notch signaling pathway‐related genes and survival

**FIGURE 1:**
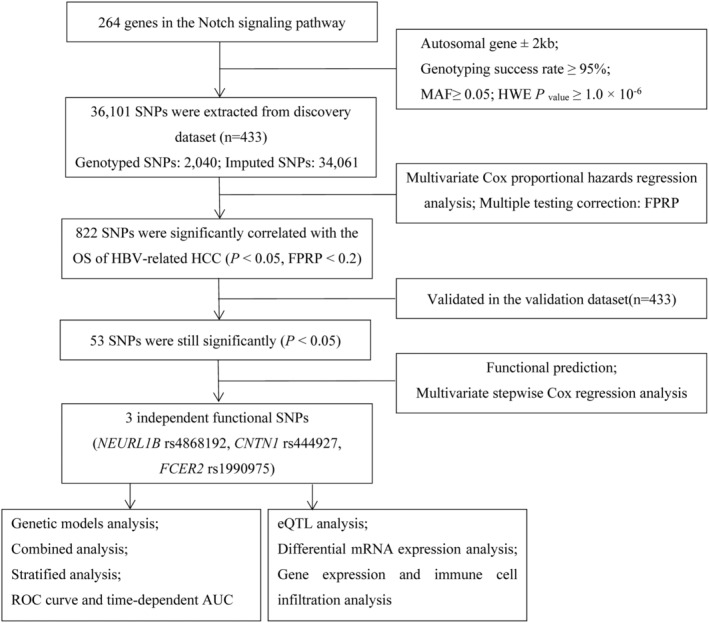
Flowchart of the study design. AUC, area under the curve; eQTL, expression quantitative trait loci; FPRP, false positive report rate; HBV, hepatitis B virus; HCC, hepatocellular carcinoma; MAF, minor allele frequency; OS, overall survival; ROC, receiver operating characteristic; SNPs, single nucleotide polymorphisms.

The analysis process of this study is shown in Figure 1. In total, 36,101 SNPs of 264 Notch signaling pathway‐related genes were obtained for analysis. In the discovery dataset, 822 SNPs were found to be significantly correlated with postoperative OS in the patients with HBV‐related HCC (*p* < 0.05, FPRP <0.2). Then, after verification in the validation dataset, 53 SNPs were still statistically significant (*p* < 0.05). Among them, 53 SNPs located on six genes (*CNTN6*, *NEURL1B*, *HDAC9*, *CNTN1*, *TLE2*, and *FCER2*; Table S2).

We used the online bioinformatics tool (SNPinfo) to predict the function of 53 SNPs. It was found that *NEURL1B* rs4868192 (T>C), *CNTN1* rs444927 (G>A), and *FCER2* rs1990975 (C>T) were transcription factors or miRNA binding sites, indicating that these three SNPs had potential functions (Table 1, Table S3). In addition, we used the RegulomeDB 2.0 and HaploReg 4.1 to predict the functions of SNPs, showing that these three SNPs could have an impact on the enhancer histone markers or motifs (Table S3). We selected the potential functional SNPs to perform multivariate stepwise Cox regression analysis in the 866 combined dataset, showing that rs4868192, rs444927, and rs1990975 were independent factors of the postoperative OS in HBV‐related HCC patients (*p* < 0.05, Table 2).

**TABLE 1:** Association between three validated significant SNPs and OS of HBV‐related HCC.

SNPs	Gene	Allele^a^	Discovery dataset (*n* = 433)				Validation dataset (*n* = 433)			Combined dataset (*n* = 866)		
			MAF	HR (95% CI)^b^	*p*	FPRP	MAF	HR (95% CI)^b^	*p*	MAF	HR (95% CI)^b^	*p*
rs4868192	*NEURL1B*	T>C	0.437	1.31 (1.07–1.6)	0.009	0.075	0.455	1.40 (1.15–1.70)	0.001	0.446	1.33 (1.16–1.53)	<0.001
rs444927	*CNTN1*	G>A	0.447	0.70 (0.57–0.86)	0.001	0.009	0.446	0.79 (0.65–0.97)	0.025	0.446	0.75 (0.65–0.86)	<0.001
rs1990975	*FCER2*	C>T	0.139	0.65 (0.48–0.88)	0.006	0.099	0.129	0.68 (0.50–0.93)	0.015	0.134	0.68 (0.55–0.85)	0.001

Abbreviations: CI, confidence interval; FPRP, false positive report rate; HBV, hepatitis B virus; HCC, hepatocellular carcinoma; HR, hazards ratio; MAF, minor allele frequency; OS, overall survival; SNPs, single nucleotide polymorphisms.

^a^ Reference allele>effect allele;.

^b^ The adjusted variables for Cox proportional hazards regression analysis were age, sex, smoking, drinking, AFP, cirrhosis, cancer embolus, and BCLC stage.

**TABLE 2:** Stepwise Cox regression analysis of clinical factors and potential functional SNPs in the combined dataset.

Variables	Cases	Multivariate analysis	
		HR (95% CI)^a^	*p* ^a^
Age (years)			
≤47	434	1	**0.023**
>47	432	**0.80 (0.65–0.97)**	
Sex			
Female	106	1	0.101
Male	760	**1.31 (0.95–1.81)**	
AFP (ng/mL)			
≤400	522	1	**0.005**
>400	344	**1.34 (1.09–1.64)**	
Cancer embolus			
No	636	1	**<0.001**
Yes	230	**1.76 (1.39–2.23)**	
BCLC stage			
0/A	427	1	**<0.001**
B/C	439	**2.01 (1.59–2.55)**	
rs4868192			
TT/TC/CC	267/426/173	**1.29 (1.12–1.48)**	**<0.001**
rs444927			
GG/GA/AA	258/443/165	**0.76 (0.66–0.88)**	**<0.001**
rs1990975			
CC/CT/TT	648/204/14	**0.68 (0.55–0.84)**	**<0.001**

Abbreviation: SNPs, single nucleotide polymorphisms.

^a^ The adjusted variables for Cox proportional hazards regression analysis were age, sex, smoking, drinking, AFP, cirrhosis, cancer embolus, and BCLC stage.

The bold value is *p* < 0.05, indicating the difference was considered statistically significant.

### 3.3 Genetic model analysis of prognosis‐related SNPs in patients with HBV‐related HCC

In the combined dataset, we found a significant correlation between the rs4868192 C allele and an increased risk of death in HCC patients, while the rs444927 A and rs1990975 T allele exhibited a protective effect in a dose–response manner (*p*_trend_ < 0.001, =0.001, and <0.001, respectively) (Table 3). The dominant genetic model showed that HBV‐related HCC patients with rs4868192 TC/CC genotypes could increase the risk of death compared with rs4868192 TT genotype (HR = 1.38, 95% CI = 1.11–1.72, *p* = 0.003), while rs444927 GA/AA genotypes and rs1990975 CT/TT genotypes had a significantly decreased risk of death compared with rs444927 GG genotype (HR = 0.76, 95% CI = 0.62–0.93, *p* = 0.009) and rs1990975 CC genotype (HR = 0.66, 95% CI = 0.52–0.83, *p* < 0.001), respectively (Table 3).

**TABLE 3:** Associations between genotypes of three independent SNPs and OS of HBV‐related HCC.

Genotypes	Cases	Deaths (%)	Multivariate analysis	
			HR (95% CI)^b^	*p* ^b^
*NEURL1B* rs4868192				
TT	267	116 (43.45)	1	–
TC	426	212 (49.77)	**1.28 (1.02–1.61)**	**0.031**
CC	173	91 (52.60)	**1.71 (1.29–2.27)**	**<0.001**
*p*_trend_	–	–	–	**<0.001**
TC/CC	599	303 (50.58)	**1.38 (1.11–1.72)**	**0.003**
*CNTN1* rs444927				
GG	258	134 (51.94)	1	–
GA	443	224 (50.56)	0.85 (0.68–1.05)	0.136
AA	165	61 (36.97)	**0.54 (0.40–0.73)**	**<0.001**
*p*_trend_	–	–	–	**<0.001**
GA/AA	608	285 (46.88)	**0.76 (0.62–0.93)**	**0.009**
*FCER2* rs1990975				
CC	648	331 (51.08)	1	**–**
CT	204	82 (40.20)	**0.66 (0.52–0.85)**	**0.001**
TT	14	6 (42.86)	0.56 (0.25–1.26)	0.161
*p*_trend_	–	–	**–**	**0.001**
CT/TT	218	88 (40.37)	**0.66 (0.52–0.83)**	**<0.001**
NPGs^a^				
0	131	77 (58.78)	1	–
1	428	212 (49.53)	**0.72 (0.55–0.93)**	**0.013**
2	256	113 (44.14)	**0.53 (0.39–0.71)**	**<0.001**
3	51	17 (33.33)	**0.34 (0.20–0.57)**	**<0.001**
Trend	–	–	**–**	**<0.001**
0	131	77 (58.78)	1	–
1–3	735	342 (46.53)	**0.61 (0.48–0.79)**	**<0.001**

Abbreviations: HBV, hepatitis B virus; HCC, hepatocellular carcinoma; NPGs, Number of protective genotypes; OS, overall survival; SNPs, single nucleotide polymorphisms.

^a^ Protective genotype: rs4868192 TT, rs444927 GA/AA, and rs1990975 CT/TT.

^b^ The adjusted variables for Cox proportional hazards regression analysis were age, sex, smoking, drinking, AFP, cirrhosis, cancer embolus, and BCLC stage.

The bold value is *p* < 0.05, indicating the difference was considered statistically significant.

### 3.4 Combined and stratified analyses of three potentially functional SNPs

**FIGURE 2:**
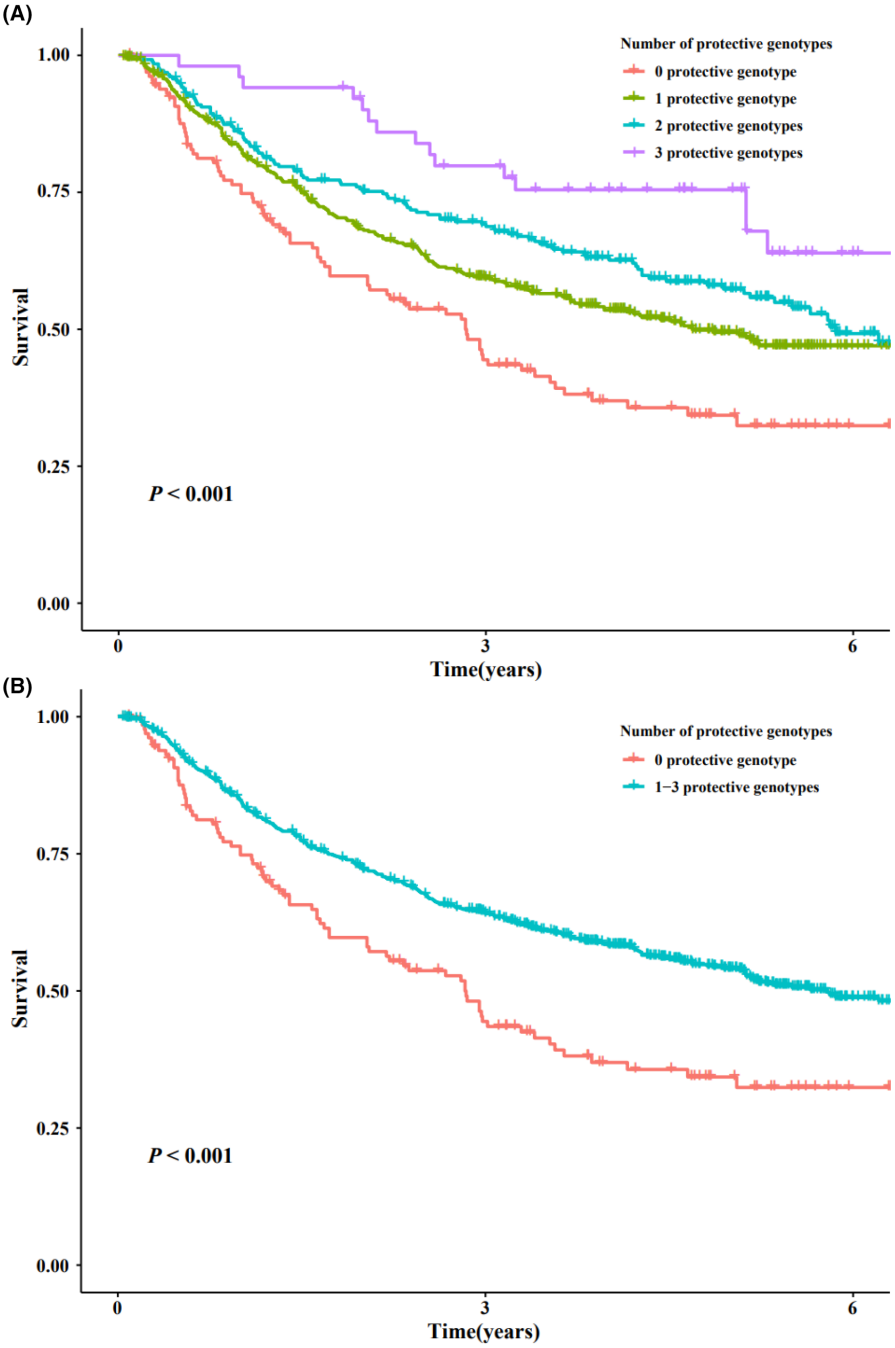
Kaplan–Meier curves of combined protective genotypes and OS of HBV‐related HCC in combined dataset. HBV, hepatitis B virus; HCC, hepatocellular carcinoma; OS, overall survival.

To further evaluate the joint effect of SNPs, we defined rs4868192 TT genotype, rs444927 GA/AA, and rs1990975 CT/TT genotypes as protective genotypes. According to the number of protective genotypes (NPGs), all patients were divided into four groups with 0, 1, 2, and 3 protective genotypes, respectively. As shown in Table 3, with the increase of the NPGs, the risk of postoperative death of patients decreased in a dose–response manner (*p*_trend_ < 0.001). Then, we divided all the patients into two groups, that is, low protective group (carrying 0 protective genotype) and high protective group (carrying 1–3 protective genotypes). It was shown that compared with the low protective group, patients in the high protective group had a better prognosis (HR = 0.61, 95% CI = 0.48–0.79, *p* < 0.001, Table 3). Kaplan–Meier curves were used to visualize the associations between the combined protective genotypes and the postoperative OS in the HCC patients (Figure 2).

In addition, we performed the stratified analysis of all clinical factors to examine the associations between combined protective genotypes in different subgroups and postoperative OS in HBV‐related HCC patients. It was shown that among subgroups of patients aged less than or equal to 47 years, older than 47 years, male, never drinking, smoking status, AFP levels, liver cirrhosis, cancer embolus, and BCLC staging, the high‐protective group had a lower risk of postoperative death than the low‐protective group (*p* < 0.05, Table 4). No interaction was found between the combined protective genotypes and clinical factors (all *p*_interaction_ > 0.05, Table 4).

**TABLE 4:** Stratified analysis of combined protective genotypes and postoperative OS of HBV‐related HCC.

Variables	0 protective genotype^a^		1–3 protective genotypes^a^		Univariate analysis		Multivariate analysis		
	Cases	Death (%)	Cases	Death (%)	HR (95% CI)	*p*	HR (95% CI)^b^	*p* ^b^	*p*_interaction_
Age^b^									
≤47	65	42 (64.62)	369	191 (51.76)	**0.56 (0.40–0.78)**	**0.001**	**0.53 (0.37–0.74)**	**<0.001**	0.184
>47	66	35 (53.03)	366	151 (41.26)	**0.65 (0.45–0.93)**	**0.020**	0.71 (0.49–1.04)	0.079	
Sex									
Female	13	6 (46.15)	93	36 (38.71)	0.72 (0.30–1.72)	0.459	0.64 (0.26–1.57)	0.329	0.245
Male	118	71 (60.17)	642	306 (47.66)	**0.60 (0.46–0.77)**	**<0.001**	**0.60 (0.46–0.78)**	**<0.001**	
Smoking									
Never	78	46 (58.97)	467	222 (47.54)	**0.60 (0.43–0.82)**	**0.002**	**0.60 (0.43–0.82)**	**0.002**	0.944
Ever	53	31 (58.49)	268	120 (44.78)	**0.61 (0.41–0.90)**	**0.013**	**0.62 (0.42 = 0.94)**	**0.023**	
Drinking									
Never	84	51 (60.71)	530	241 (45.47)	**0.54 (0.40–0.74)**	**<0.001**	**0.53 (0.39–0.72)**	**<0.001**	0.398
Ever	47	26 (55.32)	205	101 (49.27)	0.74 (0.48–1.14)	0.176	0.74 (0.48–1.16)	0.190	
AFP (ng/mL)									
≤400	81	42 (51.85)	441	190 (43.08)	**0.63 (0.45–0.88)**	**0.007**	**0.53 (0.39–0.72)**	**<0.001**	0.721
>400	50	35 (70.00)	294	152 (51.70)	**0.55 (0.38–0.79)**	**0.001**	0.79 (0.51–1.23)	0.292	
Cirrhosis									
No	57	35 (61.40)	333	149 (44.74)	**0.57 (0.39–0.72)**	**0.003**	0.70 (0.48–1.02)	0.066	0.863
Yes	74	42 (56.76)	402	193 (48.01)	**0.63 (0.45–0.88)**	**0.006**	**0.56 (0.40–0.79)**	**0.001**	
Cancer embolus									
No	90	43 (47.78)	546	217 (39.74)	**0.64 (0.46–0.89)**	**0.009**	**0.65 (0.47–0.90)**	**0.010**	0.064
Yes	41	34 (82.93)	189	125 (66.14)	**0.61 (0.41–0.89)**	**0.010**	**0.53 (0.36–0.79)**	**0.002**	
BCLC stage									
A	54	25 (46.30)	373	121 (32.44)	**0.58 (0.37–0.89)**	**0.012**	**0.57 (0.37–0.89)**	**0.012**	0.420
B/C	77	52 (67.53)	362	221 (61.05)	**0.69 (0.51–0.93)**	**0.016**	**0.66 (0.48–0.90)**	**0.008**	

Abbreviations: HBV, hepatitis B virus; HCC, hepatocellular carcinoma; OS, overall survival.

^a^ Combined protective genotypes: rs4868192 TT, rs444927 GA/AA, and rs1990975 CT/TT;

^b^ The adjusted variables for Cox proportional hazards regression analysis were age, sex, smoking, drinking, AFP, cirrhosis, cancer embolus, and BCLC stage.

The bold value is *p* < 0.05, indicating the difference was considered statistically significant.

### 3.5 ROC curves and time‐dependent AUC

**FIGURE 3:**
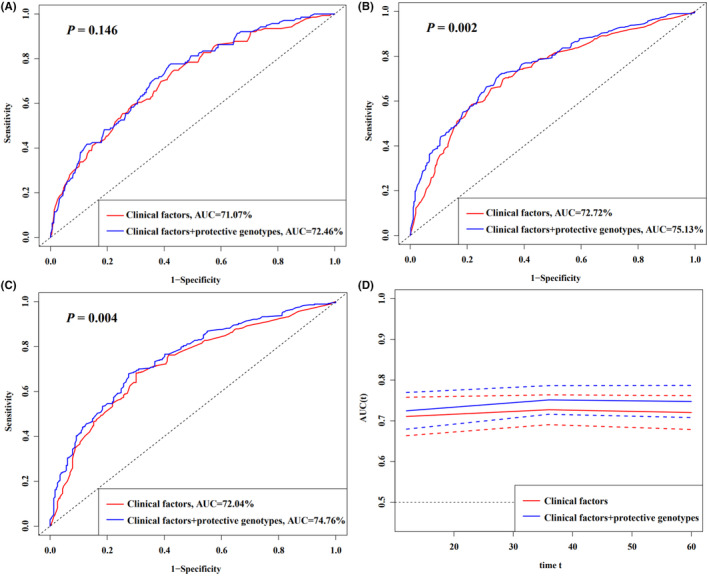
HBV‐related HCC survival prediction of the SNPs by ROC curve in combined dataset. (A–C) 1, 3, and 5 years HBV‐related HCC OS prediction by ROC curve; (D) time‐dependent AUC estimation for OS. AUC, area under the curve; HBV, hepatitis B virus; HCC, hepatocellular carcinoma; OS, overall survival; ROC, receiver operating characteristic.

Subsequently, we established predictive models to further evaluate the predictive value of combined protective genotypes in predicting the postoperative OS of the HBV‐related HCC patients. The results showed that the AUC of the model with combined protective genotypes was significantly higher than that of the model with only clinical factors (3‐year survival: 72.72% vs 75.13%, *p* = 0.002, Figure 3B; 5‐year survival: 72.04% vs 74.76%, *p* = 0.004, Figure 3C). However, the model of 1‐year survival increased the AUC from 71.07% to 72.46% without statistical significance (*p* = 0.146, Figure 3A). In addition, the time‐dependent AUC curve showed that the OS of patients with HBV‐related HCC could be predicted better by using the clinical factors and the combined protective genotypes than using only the clinical variables during follow‐up period, suggesting that the predictive model with the combined protective genotypes with clinical factors had greater predictive efficiency (Figure 3D).

### 3.6 eQTL analysis

**FIGURE 4:**
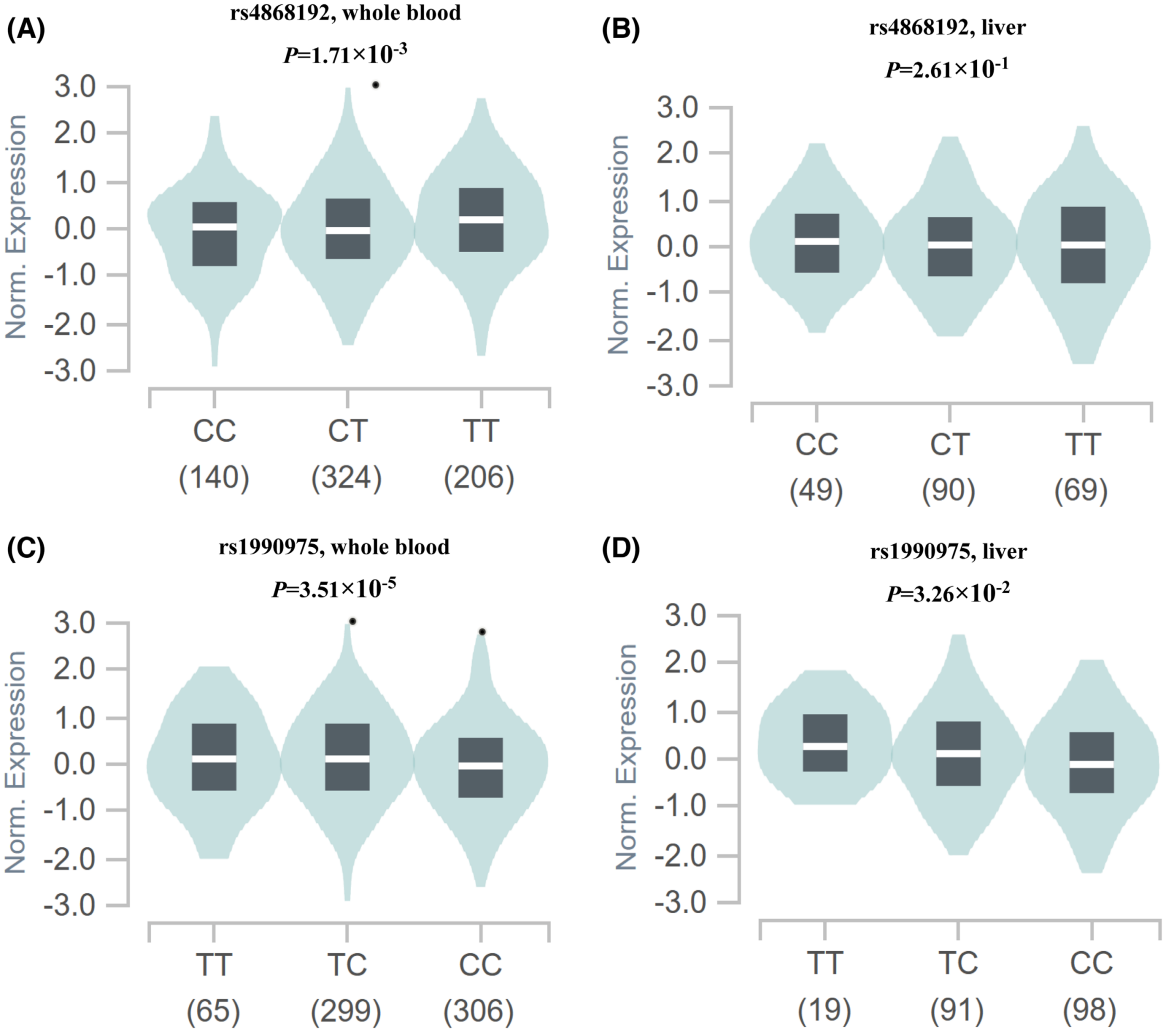
eQTL analysis of the significant SNPs and mRNA expression levels in GTEx portal. (A) NEURL1B rs4868192 in whole blood; (B) NEURL1B rs4868192 in normal liver tissue; (C) FCER2 rs1990975 in whole blood; and (D) FCER2 rs1990975 in normal liver tissue. eQTL, expression quantitative trait loci; GTEx, Genotype Tissue Expression; SNPs, single nucleotide polymorphisms.

We conducted eQTL analysis to further explore the associations between the genotypes of SNPs and the corresponding gene mRNA expression levels in normal liver tissues and the whole blood samples. The results showed that rs4868192 C allele was associated with a lower *NEURL1B* mRNA expression levels in 670 whole blood samples (*p* = 1.7 × 10^−3^, Figure 4A), the rs1990975 T allele was associated with a higher *FCER2* mRNA expression levels in 670 whole blood samples and 208 normal liver tissues (*p* = 3.5 × 10^−5^ and 0.033, respectively, Figure 4C,D). However, no correlation was found between the mRNA expression of *NEURL1B* rs4868192 and *CNTN1* rs444927 in normal liver tissues (Figure 4B; Figure S1, respectively), and there no any genotyping datasets for *CNTN1* rs444927 in the whole blood samples in GTEx portal.

### 3.7 Differential mRNA expression and survival analysis

**FIGURE 5:**
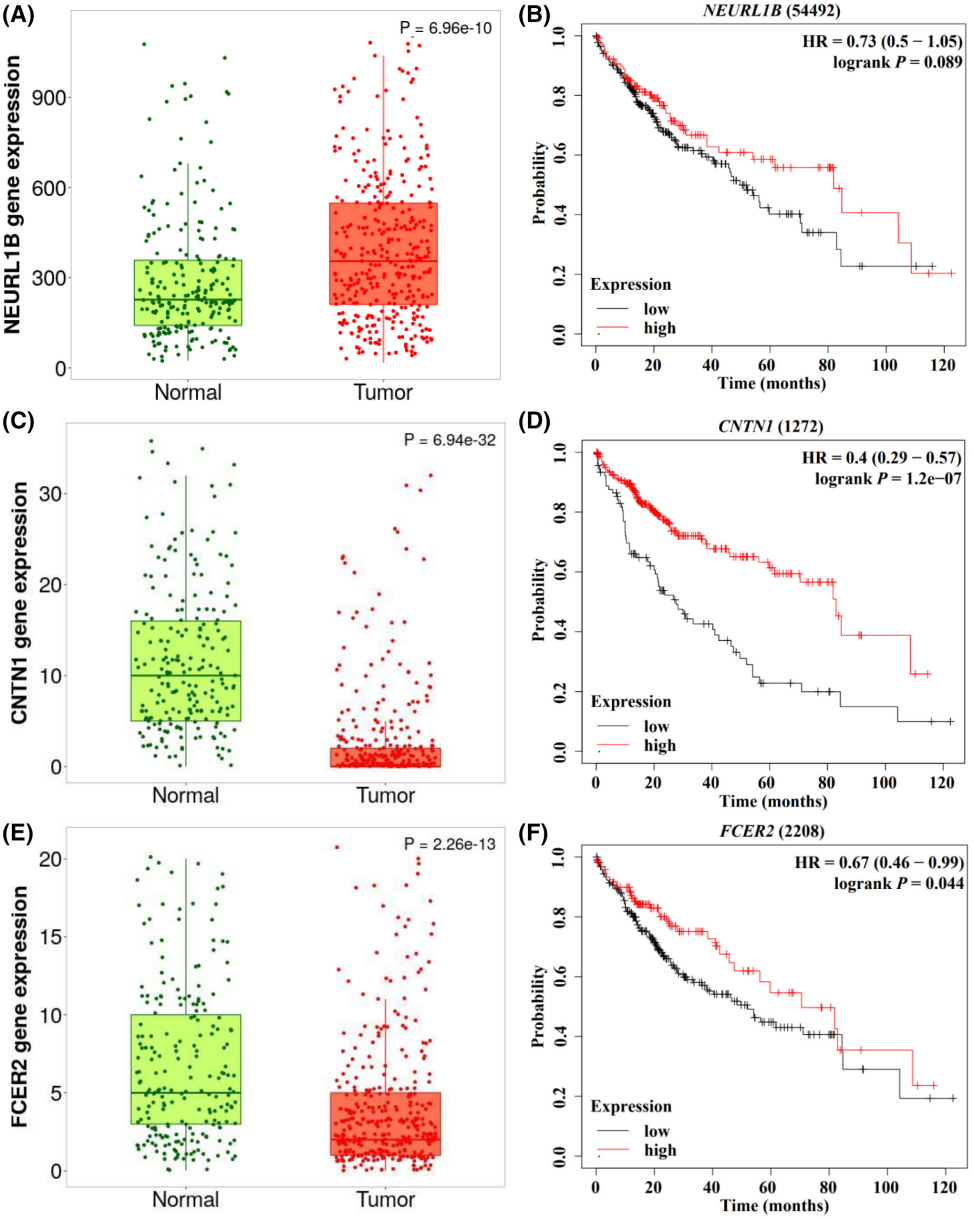
Differential mRNA expression analysis in HCC from the TNMplot database, and the associations between mRNA expression levels in tumor tissues and OS in HCC patients from Kaplan–Meier Plotter database. (A) Adjacent normal tissues versus tumor tissues: *NEURL1B*; and (B) Survival curve: *NEURL1B*; (C) adjacent normal tissues versus tumor tissues: *CNTN1* and (D) survival curve: *CNTN1*; (E) adjacent normal tissues versus tumor tissues: *FCER2* and (F) survival curve: *FCER2*. HCC, hepatocellular carcinoma; OS, overall survival.

In order to clarify the role of genes in HCC, we first compared the mRNA expression levels of the three genes based on TNMlot database. We found that the mRNA expression levels of *NEURL1B* was increased, while the mRNA expressions levels of *CNTN1* and *FCER2* were decreased in the HCC tissues compared with the normal tissues (*p* = 6.96 × 10^−10^, 6.94 × 10^−32^ and 2.26 × 10^−13^, respectively, Figure 5). In addition, we evaluated the correlation between the gene mRNA expression levels and the HCC survival based on the Kaplan–Meier Plotter database, showing that higher mRNA expression level of *NEURL1B*, *CNTN1*, and *FCER2* were associated with a better OS (HR = 0.73, 95% CI = 0.5–1.05, *p* = 0.089; HR = 0.4, 95% CI = 0.29–0.57, *p* = 1.2 × 10^−7^; HR = 0.67, 95% CI = 0.46–0.99, *p* = 0.044, respectively, Figure 5).

### 3.8 Gene expression and immune cell infiltration analysis

We used the TIMER2.0 to analyze the association between the expression levels of gene mRNA and the immune cell infiltration levels in the tumor microenvironment of HCC. As shown in Figure S2, the mRNA expression levels of *NEURL1B*, *CNTN1*, and *FCER2* were positively correlated with a variety of immune cells in the tumor microenvironment (CD4^+^ T cell: R/*p* = 0.251/2.28 × 10^−6^, 0.193/3.17 × 10^−4^, and 0.177/9.45 × 10^−4^, respectively; CD8^+^ T cell: R/*p* = 0.21/8.39 × 10^−5^, 0.142/8.36 × 10^−3^, and 0.14/9.18 × 10^−3^, respectively; Tregs cell: R/*p* = 0.292/3.23 × 10^−8^, 0.257/1.28 × 10^−6^, and 0.146/ 6.46 × 10^−3^, respectively; B cell: R/*p* = 0.183/6.34 × 10^−4^, 0.173/1.29 × 10^−3^, and 0.382/2.05 × 10^−13^, respectively).

## 4 DISCUSSION

In this study, we conducted a two‐stage analysis to investigate associations between SNPs of Notch signaling pathway genes and OS of HBV‐related HCC. We found that the *NEURL1B* rs4868192 T>C, *CNTN1* rs444927 G>A, and *FCER2* rs1990975 C>T, located at miRNA or transcription factor binding sites, were associated with OS. Meanwhile, the rs4868192 C allele was correlated to lower mRNA expression levels of *NEURL1B*, and the rs1990975 T was correlated to higher mRNA expression levels of *FCER2*, likely affecting the functions of the gene. Furthermore, we found that the expression levels of *NEURL1B* mRNA were higher, and the expression levels of *CNTN1* and *FCER2* mRNA were lower in the HCC tissues, and these high mRNA expression levels correlated with a better survival of HCC. These results implied that these potentially functional SNPs could serve as prognostic biomarkers for the HBV‐related HCC.

In mammals, Neur includes two homologs: neuralized E3 ubiquitin protein ligase 1 (*NEURL1*) and neuralized E3 ubiquitin protein ligase 1B (*NEURL1B*, also known as *NEURL3*), both of which could interact with Notch ligands Delta‐like 1 and Jagged1, thereby regulating the Notch pathway.^22^, ^23^ *NEURL1B* also acted as the E3 ubiquitin ligase of IRF7, which was selectively upregulated in the late stage of host immune response. By ubiquitination of the K63‐linked poly‐ubiquitin chain on IRF7, *NEURL1B* enhanced IRF7‐mediated transcription of interferon‐stimulated genes by increasing the H3K27ac signal in the IRF7‐binding regions to augment the host antiviral immune response.^24^ Previous research indicated that *NEURL1B* was low expressed in colon cancer, and its low expression predicted a shorter survival time, suggesting that *NEURL1B*, as a tumor suppressor gene, participated in the pathological processes of colon cancer and may be served as potential biomarkers for early diagnosis and prognostic evaluation.^25^ In this study, we found that the *NEURL1B* rs4868192 C allele could increase the risk of death in patients with HBV‐related HCC, likely through downregulating expression levels of *NEURL1B* mRNA. Interestingly, *NEURL1B* was highly expressed in the HCC tissues, while its lower mRNA expression levels were associated with worse survival, which could be attributed to the decreased levels of immune cell infiltration in the tumor microenvironment.

Contact protein 1 (*CNTN1*), located on chromosome 12q12, is a protein coding gene that encodes members of the immunoglobulin superfamily. This superfamily is a glycosyl phosphatidylinositol anchored nerve cell adhesion molecule that acts as a cell adhesion molecule.^26^ *CNTN1* also serves as a functional ligand of Notch1 and promotes Notch1 activation by releasing the Notch intracellular domain (NCID), thereby promoting tumor progression.^27^, ^28^ Recently, *CNTN1* has been reported to mediate cell functions involving multiple signaling pathways, including the Notch signaling pathway, RHOA dependent pathway, RET/PTC3 pathway, and VEGFC/FLT4 axis to promote the invasion and metastasis of cancer cells.^29^, ^30^ *CNTN1* is commonly considered as a oncogenic gene and overexpress in multiple cancers. For example, in HCC, overexpression of CNTN1 is closely related to multiple malignant phenotypes and poor prognosis.^31^ Another study demonstrated that *CNTN1* was upregulated in thyroid cancer, and knocking down of *CNTN1* could inhibit the proliferation, migration, and invasion through regulating the Notch pathway.^30^ In contrast to the above research results, Candelaria Bracalente et al. found that A7 could upregulate *CNTN11*, which was involved in melanocyte differentiation and axon guidance, leading to higher differentiation and lower invasion of melanoma.^32^ In this study, we found that *CNTN1* rs444927 A allele could decrease the risk of death in the patients with HBV‐related HCC after hepatectomy, and the *CNTN1* mRNA expression levels in the HCC tissues was decreased compared with the adjacent normal tissues, and higher expression levels were associated with better survival. Unfortunately, we did not find any correlation between the rs444927 A allele and *CNTN1* mRNA expression levels.

Fc epsilon receptor II (*FCER2*), also named as *CLEC4J*, is a protein coding gene located on chromosome 19p13.3 and expressed in various cells such as B cells, eosinophils, follicular dendritic cells, macrophages, and platelets.^33^ *FCER2* plays essential roles in the growth and differentiation of T and B cells, the production and response of IgE, cell adhesion, and antigen presentation.^33^, ^34^ It has been shown that the expression levels of *FCER2* in HCC tissues was significantly lower than that in normal liver tissues. In addition, *FCER2* was significantly associated with individual cancer stages, and the expression of *FCER2* decreases with tumor progression.^35^ Another study reported that the expression of *FCER2* was significantly decreased in prostate cancer, which could serve as a reliable biomarker to avoid unnecessary prostate biopsies and misdiagnosis.^36^ In a nasopharyngeal carcinoma study, it was found that *FCER2* was a downregulated gene in nasopharyngeal carcinoma, and its expression levels were significantly lower in nasopharyngeal carcinoma than in adjacent tissues. This gene was the first confirmed diagnostic marker related to immune cell infiltration in nasopharyngeal carcinoma.^37^ Our study indicated that *FCER2* rs1990975 T allele may reduce the risk of death in the HBV‐related HCC patients by upregulating the mRNA expression levels of *FCER2*. In addition, the expression levels of *FCER2* mRNA were decreased in the HCC tissues and higher mRNA expression levels were associated with a better survival. Consistent with the previous studies, these findings suggested that the *FCER2* may participate in the progress of HCC as a tumor suppressor gene.

In conclusion, we identified three potentially functional SNPs (*NEURL1B* rs4868192 T>C, *CNTN1* rs444927 G>A, and *FCER2* rs1990975 C>T), which were significantly associated with HBV‐related HCC OS, likely by regulating the mRNA expression of the corresponding genes. Once validated, genetic variants in Notch signaling pathway‐related genes may serve as new potential prognostic biomarkers of the HBV‐related HCC. However, there are limitations in this study. Firstly, we did not have detailed treatment information on the surgical patient population, which did not allow further stratified analyses; secondly, participants were from Southern China only, and the sample size was limited; therefore, the findings may not generalizable to the other patient populations; thirdly, some of the mRNA expressions do not seem to be affected by the SNPs examined, this is likely because their effects were too small to be detected by the GTEx dataset; and finally, the biological mechanisms underlying these identified associations remain unclear, and additional functional experiments need to be performed in the future.

## AUTHOR CONTRIBUTIONS

**Liming Qin:** Formal analysis (equal); investigation (equal); writing – original draft (equal). **Moqin Qiu:** Formal analysis (equal); investigation (equal). **Qiuling Lin:** Investigation (equal). **Binbin Jiang:** Investigation (equal). **Shicheng Zhan:** Investigation (equal). **Xueyan Wei:** Investigation (equal). **Junjie Wei:** Investigation (equal). **Yingchun Liu:** Data curation (equal); investigation (equal). **Qiuping Wen:** Software (equal). **Peiqin Chen:** Software (equal). **Yanji Jiang:** Investigation (equal). **Zihan Zhou:** Investigation (equal). **Xiumei Liang:** Investigation (equal). **Ji Cao:** Investigation (equal). **Yizhen Gong:** Software (equal). **Yuying Wei:** Software (equal). **Xiaoxia Wei:** Software (equal). **Hongping Yu:** Conceptualization (equal); funding acquisition (equal); resources (equal); writing – review and editing (equal).

## FUNDING INFORMATION

This study was supported by Key Laboratory of Early Prevention and Treatment for Regional High Frequency Tumor, Ministry of Education (GKE‐ZZ202104); Key Laboratory of Early Prevention and Treatment for Regional High Frequency Tumor, Ministry of Education (GKE‐ZZ202118); Key Laboratory of Early Prevention and Treatment for Regional High Frequency Tumor, Ministry of Education (GKE‐KF202102); Guangxi Medical and health appropriate Technology opening and popularization and application project (S2021022); and Youth Program of Scientific Research Foundation of Guangxi Medical University Cancer Hospital (YQJ2022‐5).

## CONFLICT OF INTEREST STATEMENT

The authors have no conflict of interest.

## ETHICS STATEMENT

Approval of the research protocol by an Institutional Reviewer Board: This study was conducted according to the guidelines of the Declaration of Helsinki, and approved by Institutional Review Committee of the Guangxi Medical University Cancer Hospital (LW2023052).

## CONSENT

Informed consent was obtained from all subjects involved in this study.

## Supporting information


Figure S1.
Figure S2.
Table S1.
Table S2.
Table S3.

## Data Availability

The data that support the findings of this study are available from the corresponding author upon reasonable request.
