# Supplementary material for: Association between novel genetic variants of Notch signaling pathway genes and survival of hepatitis B virus‐related hepatocellular carcinoma
Source: Cancer Med. 2024 Apr 1;13(7):e7040. doi: 10.1002/cam4.7040 (PMC10985410; doi:10.1002/cam4.7040)
Supplement: Supplementary file 1 — Figure S1. Figure S2. Table S1. Table S2. Table S3. [file CAM4-13-e7040-s001.docx]

**Supporting Information**

**Supplementary figure 1:**

**eQTL analysis of rs444927 in GTEx protal.** (A) *CNTN1* rs444927 in normal liver tissue.


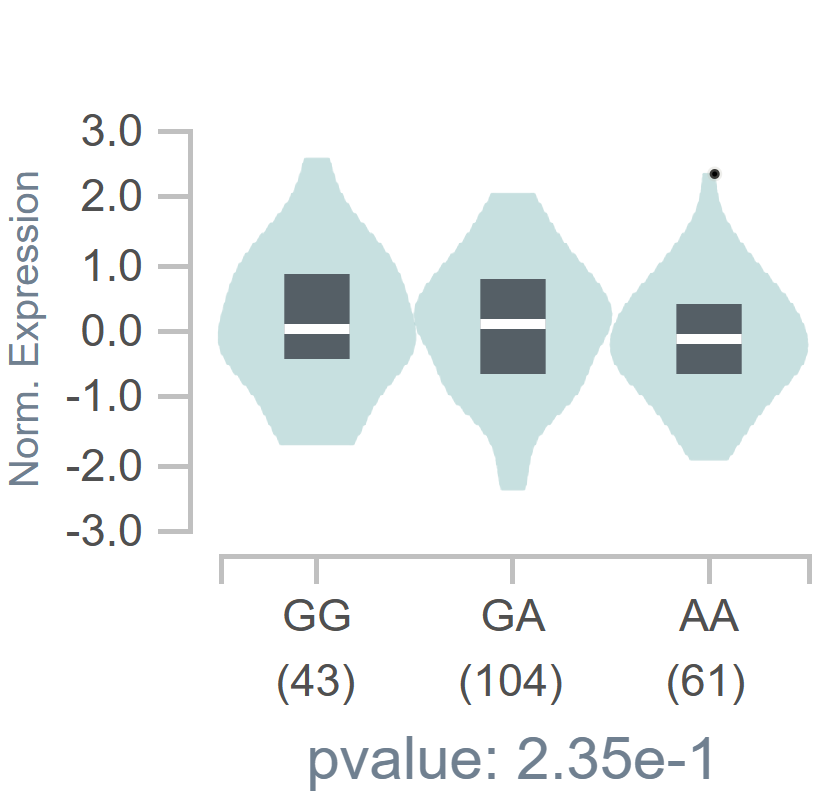


**rs444927, liver**

***P*=2.35×10^-1^**

**A**

**rs444927, liver**

***P*=2.35×10^-1^**

**Supplementary** **figure 2:**

**Gene expression and immune cell infiltration analysis.** (A) - (D) *NEURL1B*; (E) - (H) *CNTN1*; (I) - (L) *FCER2*.

**B**

**A**


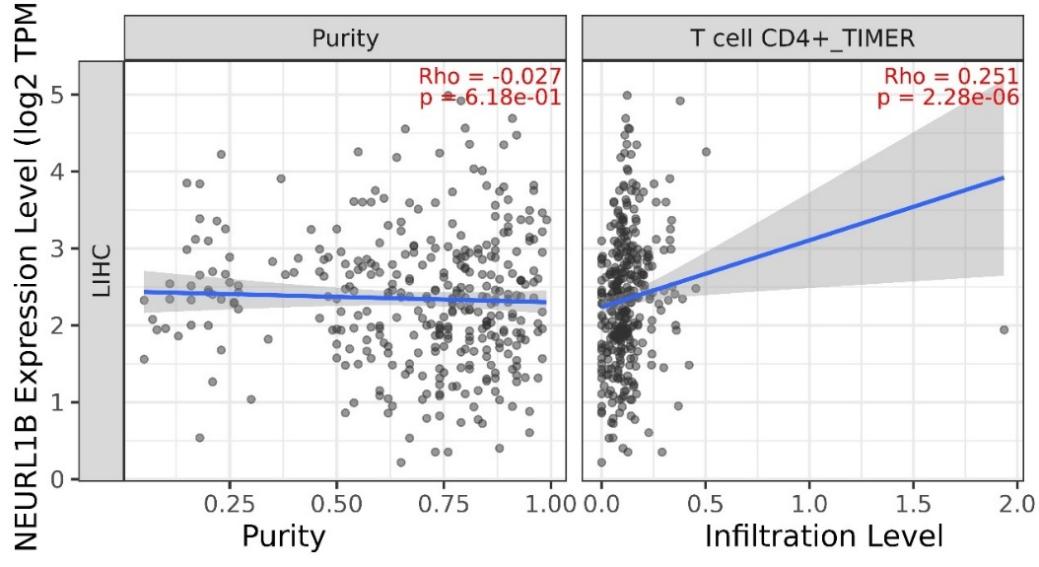

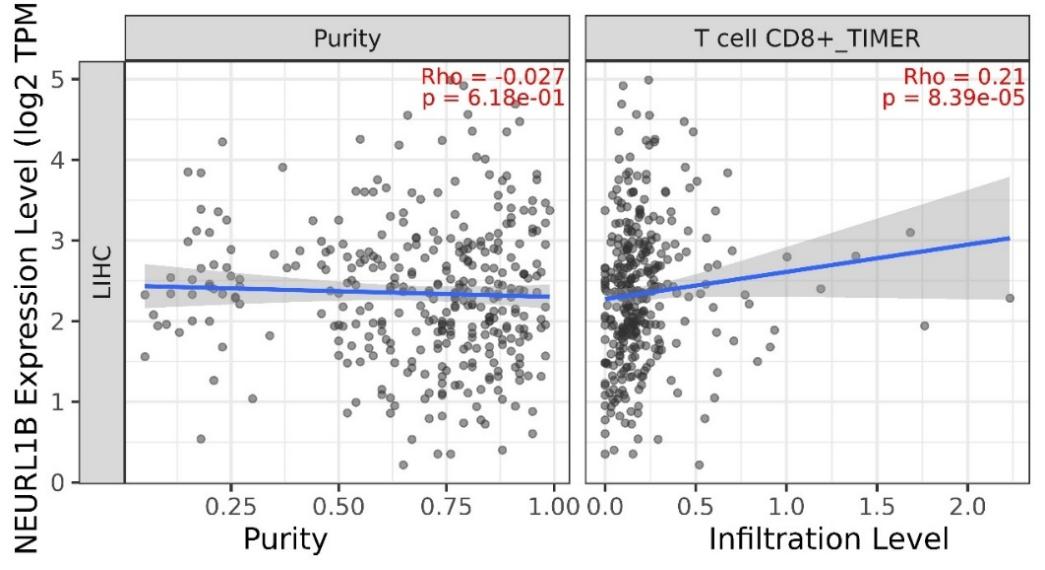


**C**


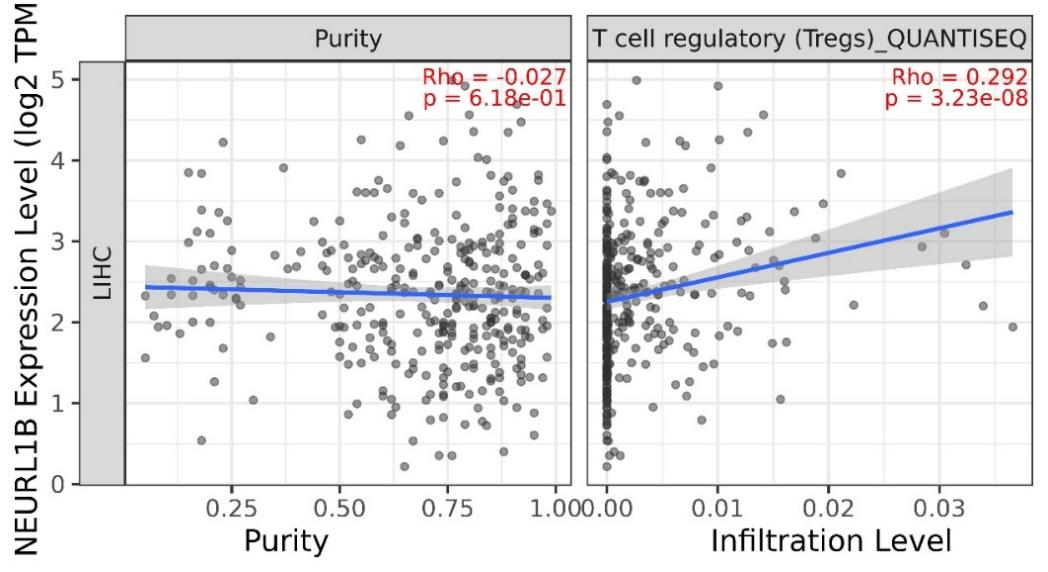

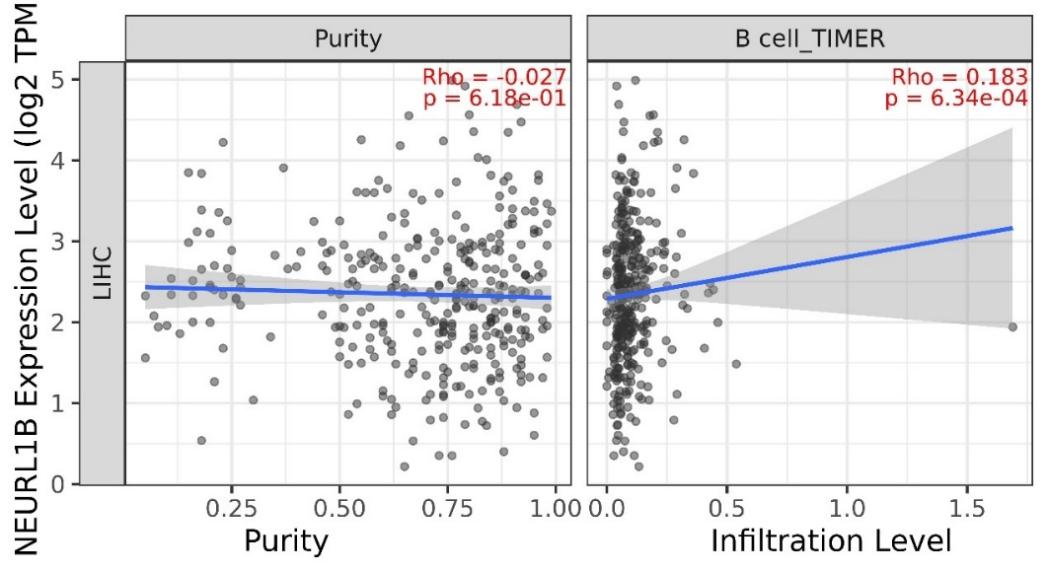


**D**

**E**


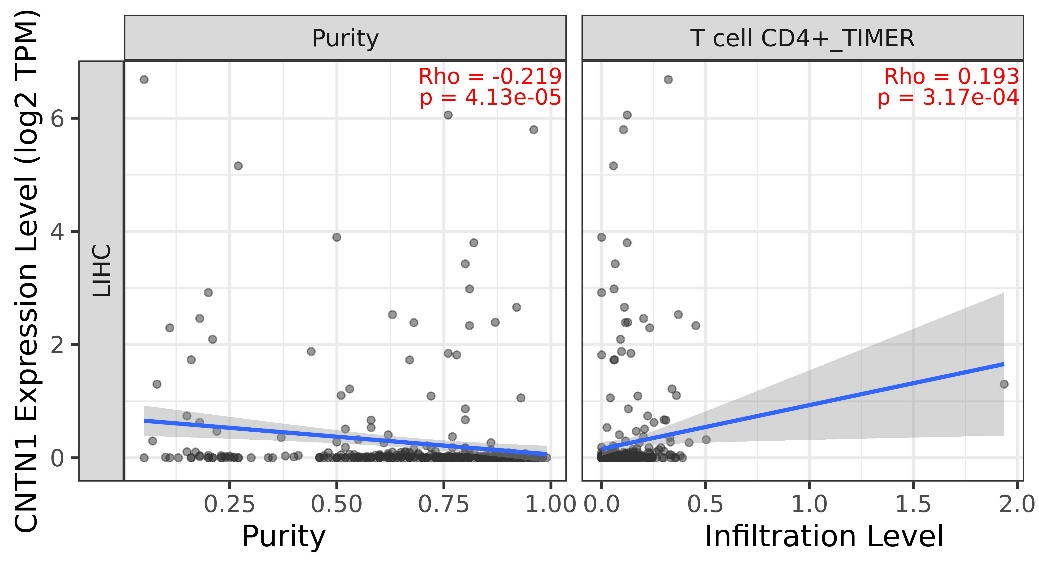

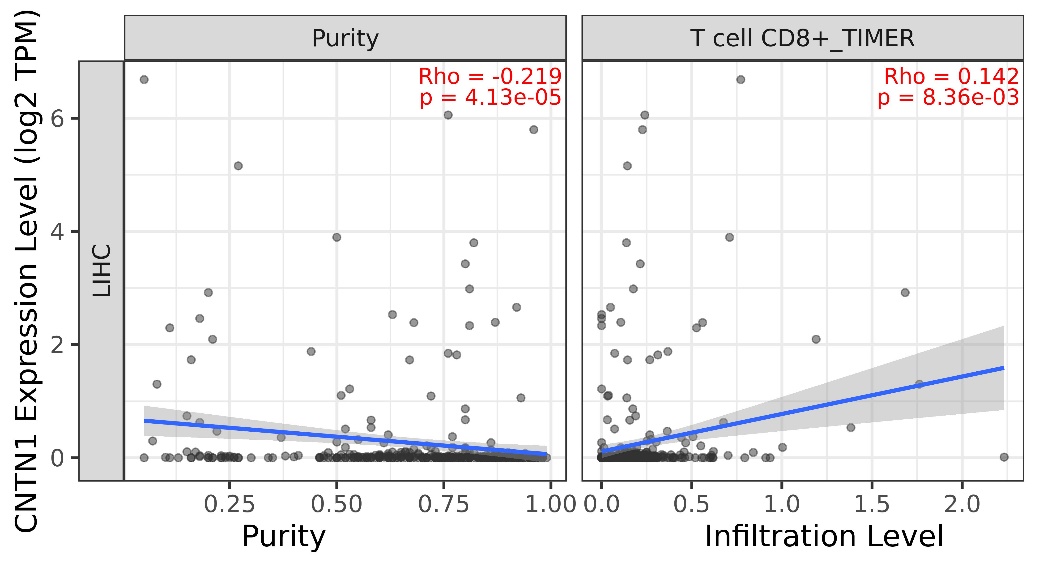


**G**

**F**


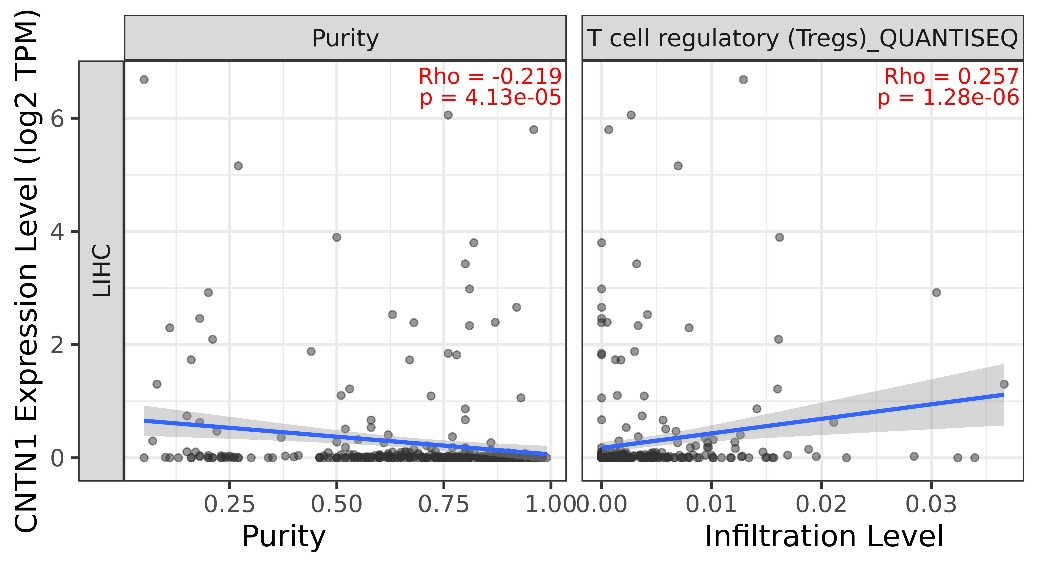

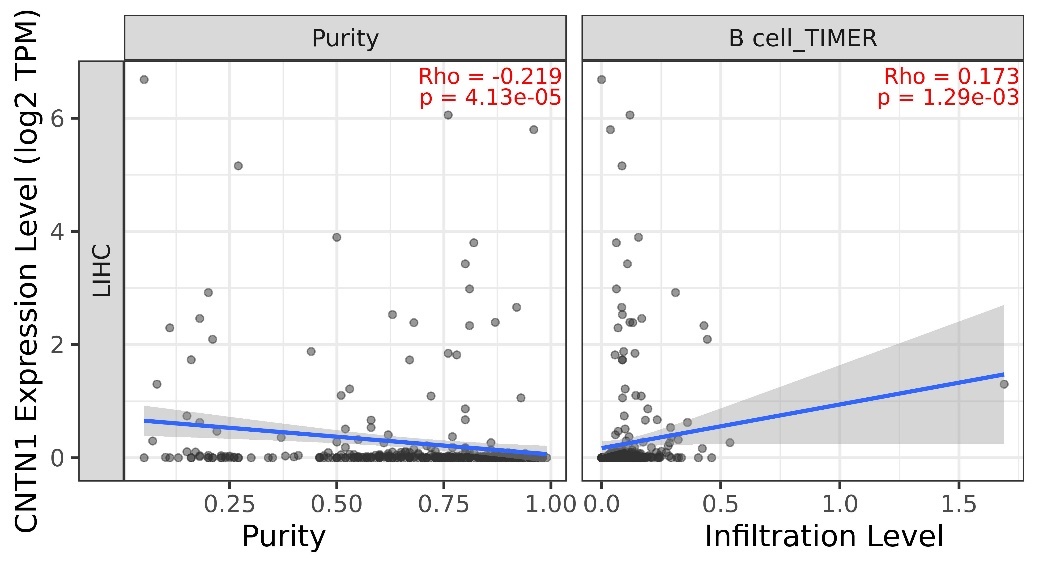


**H**

**J**

**I**


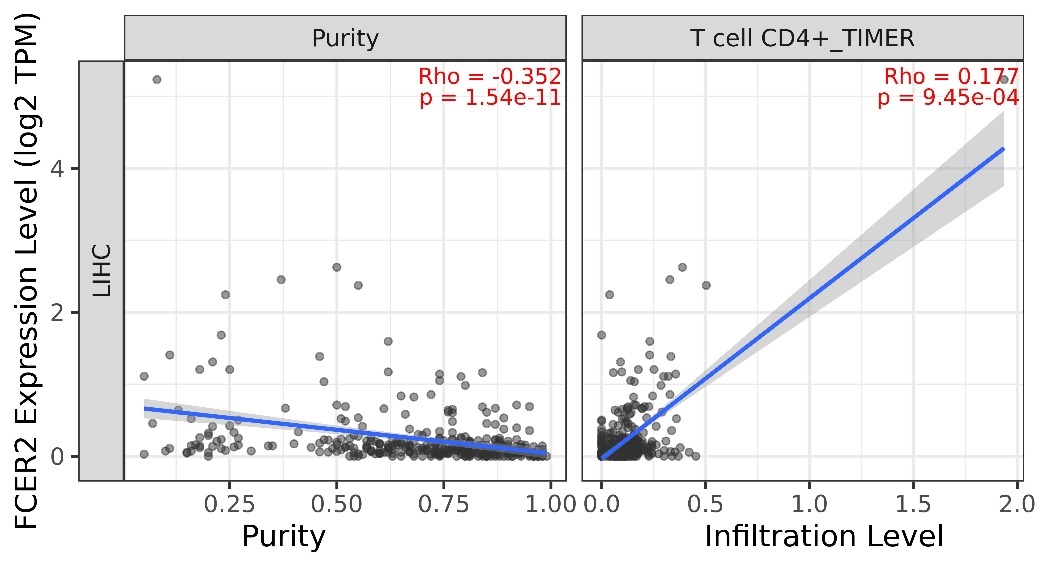

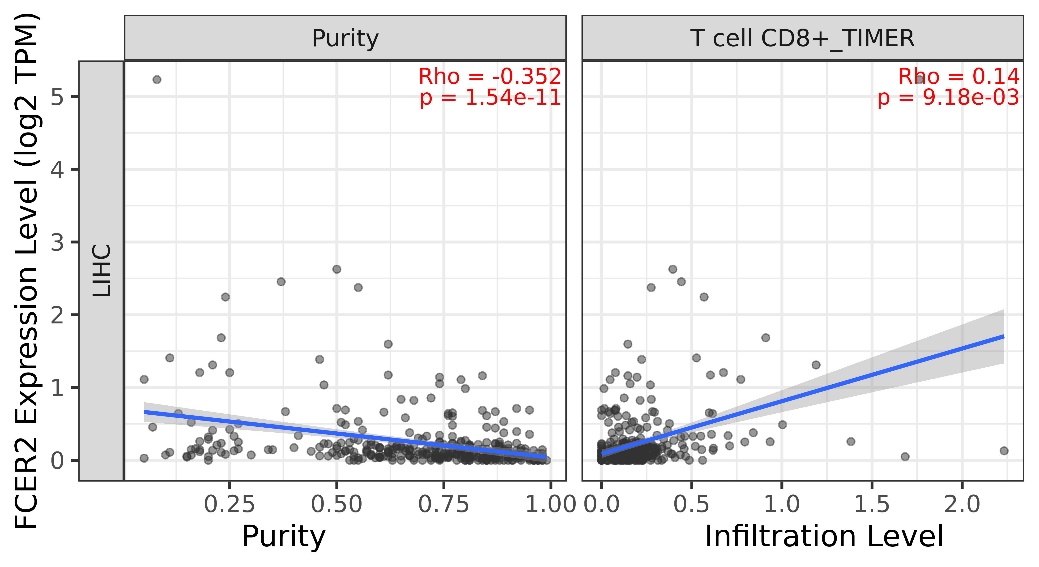


**K**


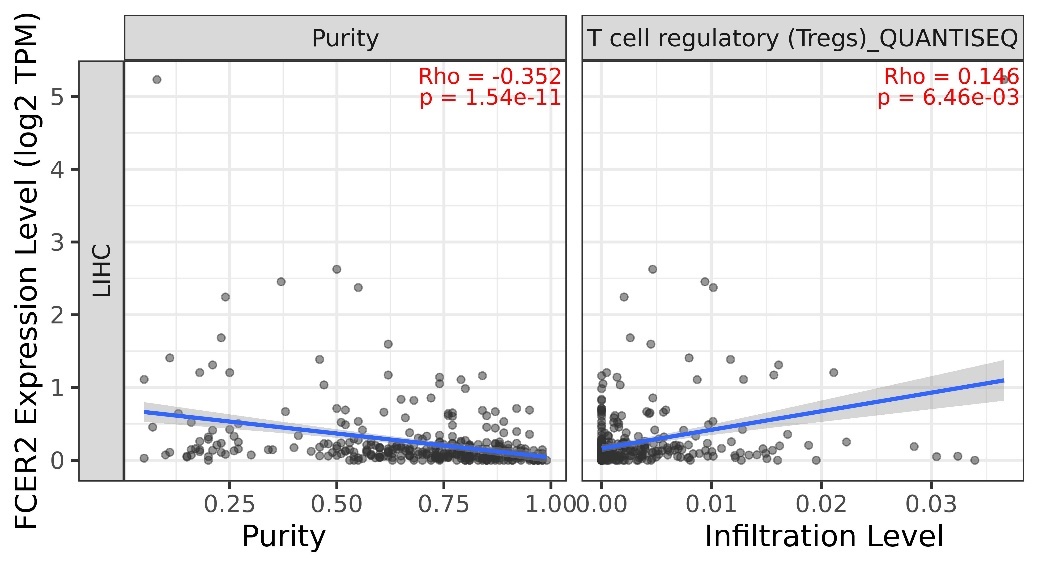

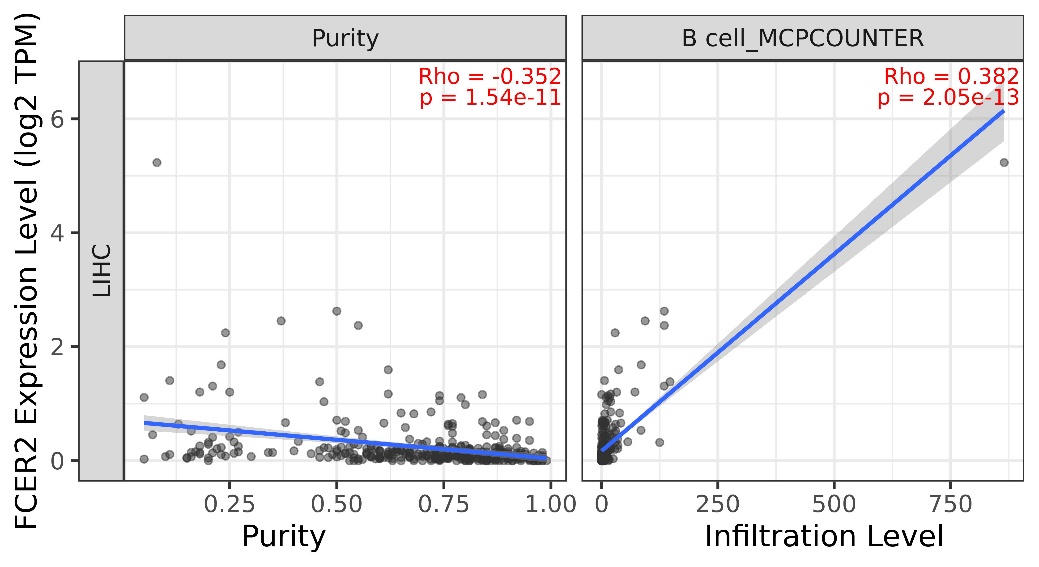


**L**

**Supplementary table 1:**

**List of 264 Notch pathway genes from MsigDB and PathCards**

| Dataset | Name of pathway ^a^ | Selected genes | Number of genes |
| --- | --- | --- | --- |
| BIOCARTA | NOTCH_PATHWAY | *PSEN1, FURIN, ADAM17, RBPJ, DLL1, NOTCH1* | 6 |
| KEGG | NOTCH_SIGNALING_PATHWAY | *DLL3, RBPJL, DTX2, CREBBP, CTBP1, CTBP2, DTX3L, PTCRA, JAG1, DTX1, DVL1, DVL2, DVL3, DTX3, EP300, SNW1, DTX4, NCSTN, KAT2A, DLL1, HDAC1, HDAC2, HES1, RBPJ, JAG2, HES5, LFNG, MFNG, NOTCH1, NOTCH2, NOTCH3, NOTCH4, APH1A, DLL4, MAML3, PSENEN, PSEN1, PSEN2, RFNG, ADAM17, MAML2, NUMB, KAT2B, NUMBL, CIR1, NCOR2, MAML1* | 47 |
| PID | NOTCH_PATHWAY | *SSPOP, DLL1, ADAM10, ADAM12, KDM1A, NCOR1, NEURL1, MYC, IL4, ENO1, FURIN, CBL, GATA3, CCND1, YY1, CDKN1A, EPS15, NOTCH1, NUMB, PSEN1, MFAP2, RAB11A, SKP1, JAG1, DLK1, NOTCH2, DNM1, RBPJ, EP300, CNTN1, SKP2, MFAP5, CTBP1, HDAC1, CUL1, PTCRA,MARK2, DTX1, MIB1, MAML2, DNER, LNX1, APH1B, NCSTN, MAML1, FBXW7, APH1A, ITCH, SPEN, MYCBP, NOTCH4, RBBP8, DLL4, DLL3, PSENEN, NOTCH3, CNTN6, JAG2, NCOR2* | 59 |
| REACTOME | SIGNALING_BY_NOTCH | *PLXND1, CREBBP, PSMB1, PSMC4, MAMLD1, TACC3, CNTN1, FLT4, PSMA4, HDAC9, CUL1, HDAC7, ST3GAL6, TLE2, YBX1, HDAC4, SEL1L, NOTCH3, ATP2A3, SIRT6, TNRC6C, ITCH, PSEN1, B4GALT1, TMED2, PSMC5, TNRC6A, DTX2, PSME1, AGO1, HDAC6, PSMD5, PSMD8, MFNG, TNRC6B, RBX1, EP300, HDAC10, GZMB, PSMC6, PSMA3, SNW1, HIF1A, PSMC1, PSMB5, PSMA6, PSME2, PSMA7, POFUT1, JAG1, E2F1, MIB1, PSMD10, TBL1X, PSMD7, FCER2, H2AZ2, LFNG, PSMA2, TLE4, ACTA2, NEURL1, PSMD3, PSMD11, KAT2A, HDAC5, FBXW7, DTX4, ST3GAL4, CCND1, MDK, PSMD9, CCNC, E2F3, SKP1, WWC1, TFDP2, KAT2B, HES1, PSMD14, STAT1, HDAC1, APH1A, CREB1, AGO2, H2BC11, PSMF1, PSMB2, AGO3, ST3GAL3, DLGAP5, SEM1, DLL4, PSMA1, PSME3, H3-3B, CDK8, NUMB, NOTCH2, AGO4, DTX1, HEY2, PSMB7, MYC, ARRB1, ADAM10, APH1B, EGF, TLE3, FURIN, NCOR1, ARRB2, TP53, AKT1, PSMB6, PSMA5, PSEN2, RPS27A, H2BC1, EGFR, HDAC8, NOTCH1, UBC, ADAM17, MOV10, H2BC5, H4C8, RUNX1, PSMD4, PSMB4, MAML1, PSMC2, NCSTN, H3-3A, POGLUT1, ELF3, HDAC11, PRKCI, PSMD6, HEYL, H2AZ1, FABP7, HEY1, YWHAZ, PSMC3, SMAD3, RBPJ, RFNG, UBB, PTCRA, HDAC3, NBEA, PSMD1, ATP2A2, PSMD2, RAB6A, TBL1XR1, JUN, H2AC6, H2BC4, H3C13, H2AC20, MAML2, H2BC21, JAG2, H2BC13, DLK1, PSMD13, PBX1, KZF1, DNER, H2AX, ATP2A1, NCOR2, HDAC2, TLE1, MAML3, H2AC7, H2BU1, H4C3, H3C12, PSMD12, H4C11, H3C4, MIB2, H4-16, H2BC12, HES5, TFDP1, WWP2, DLL1, MIR449A, H3C14, H3C15, PSMB8, NOTCH4, PSENEN, PSMB10, MIR34C, MIR206, MIR181C, MIR200C, MIR449B, MIR200B, MIR150, MIR34B, MIR302A, NEURL1B, UBA52, H2BC15, H2BS1, PSMB9, H2AJ, MIR449C, H4C15, H4C14, H4C12, H2BC14, H2BC8, H3C8, H2AB1, H2BC6, H4C6, H2BC17, H3C6, H4C13, H3C11, H2BC9, H3C1, PSMB3, H4C9, H2AC14, H2BC3, H4C5, H2AC8, H4C4, H2BC7, H3C7, H2AC4, H2BC10, H4C1, H4C2, H3C10, H3C2, H3C3* | 245 |
| WP | NOTCH_SIGNALING_PATHWAY | *MIR1281, ENSG00000284930, CDKN1A, DLL3, PTCRA, JAG1, DTX1, EP300, AKT1, FHL1, SNW1, SPEN, NCSTN, HEY1, HEY2, GATA3, DLL1, GSK3B, HDAC1, HDAC2, HIF1A, HES1, RBPJ, JAG2, JAK2, HES5, LCK, MAGEA1, MAPT, MYC, NFKB1, NOTCH1, NOTCH2, NOTCH3, NOTCH4, APH1A, PIK3R1, PIK3R2, DLL4, FBXW7, HES6, MAML3, PSENEN, PSEN1, PSEN2, CCND1, RING1, SKP1, SRC, STAT3, ADAM17, TLE1, APH1B, ITCH, MAML2, CUL1, NUMB, SAP30, NUMBL, CIR1, NCOR1, NCOR2, MAML1* | 63 |
| PachCards | Notch Signaling Pathways | *MIB1, NOTCH3, PSENEN, RFNG, PIK3R2, RBPJ, DYRK1A, CIR1, HES1, EPN1, NOTCH4, CCN3, BCL2, AKT1, NRARP, CSNK2A1, NCOR1, HEY1, CLTC, DLL1, DLK1, IKBKG, MTOR, TACC3, NLK, SAP30, NOTCH2, APH1A, MFNG, PIK3CG, H4C16, CDK8, CTBP1, AP2A1, NOTCH1, NCSTN, FURIN, PIK3R1, H3-3A, MAML3, BEND6, THBS4, DNM3, PSEN2, CTNNB1, PIK3CD, EHMT2, MAML2, BCL6, NUMB, DNM1, ADAM17, NTN1, NFKB2, SNW1, KAT2A, HIF1A, HDAC2, PICALM, DLL4, MFAP5, NFKB1, FBXW7, TAF6L, SMAD3, HDAC1, DNM2, PSEN1, THBS2, PIK3CB, EHMT1, MAML1, KDM1A, SIRT1, EPN3, JAG2, CNTN6, IKBKB, AKT3, RUNX3, CSNK2B, NKAP, CCND1, EPN2, JAG1, CNTN1, CHUK, AKT2, RUNX2, CSNK2A2, NCOR2, MYC, CLTCL1, DLL3, DNER, IKBKE, POFUT1, EP300, SMAD1, SPEN* | 100 |
| Exclude |  | 184 duplicated genes, 63 genes are not in the hg19 genome reference and 9 genes on the X chromosome (*MAMLD1, HDAC6, PSMD10, TBL1X, HDAC8, FHL1, MAGEA1, IKBKG, NKAP*) | 256 |
| All |  |  | 264 |

^a^ Genes were selected based on online datasets;

Keyword: notch;

Organism: Homo sapiens

**Supplementary table 2:**

**Association between 53 Notch pathway gene SNPs and OS of HBV-related HCC**

| SNPs | Gene | Allele ^a^ | Discovery dataset (n=433) | | | | Validation dataset (n=433) | | | Combined dataset (n=866) | | |
| --- | --- | --- | --- | --- | --- | --- | --- | --- | --- | --- | --- | --- |
|  |  |  | MAF | HR (95%CI) ^b^ | *P* | FPRP | MAF | HR (95%CI) ^b^ | *P* | MAF | HR (95%CI) ^b^ | *P* |
| rs1394174 | *CNTN6* | A>G | 0.491 | 0.79 (0.65-0.97) | 0.028 | 0.188 | 0.480 | 0.82 (0.68-0.99) | 0.034 | 0.495 | 1.00 (0.87-1.14) | 0.966 |
| rs10035202 | *NEURL1B* | T>C | 0.437 | 1.29 (1.06-1.58) | 0.011 | 0.118 | 0.458 | 1.41 (1.16-1.72) | 0.001 | 0.448 | 1.33 (1.16-1.53) | <0.001 |
| rs4868192 | *NEURL1B* | T>C | 0.437 | 1.31 (1.07-1.6) | 0.009 | 0.075 | 0.455 | 1.40 (1.15-1.70) | 0.001 | 0.446 | 1.33 (1.16-1.53) | <0.001 |
| rs35254338 | *NEURL1B* | C>T | 0.463 | 1.3 (1.07-1.59) | 0.009 | 0.095 | 0.476 | 1.39 (1.14-1.71) | 0.001 | 0.469 | 1.31 (1.14-1.51) | <0.001 |
| rs4867682 | *NEURL1B* | T>C | 0.454 | 1.29 (1.06-1.58) | 0.012 | 0.118 | 0.470 | 1.37 (1.11-1.68) | 0.003 | 0.462 | 1.29 (1.12-1.49) | <0.001 |
| rs11749132 | *NEURL1B* | G>A | 0.454 | 1.29 (1.06-1.58) | 0.012 | 0.118 | 0.470 | 1.37 (1.11-1.68) | 0.003 | 0.462 | 1.29 (1.12-1.49) | <0.001 |
| rs11745123 | *NEURL1B* | C>T | 0.468 | 1.3 (1.07-1.59) | 0.009 | 0.095 | 0.492 | 1.22 (1.00-1.48) | 0.047 | 0.480 | 1.23 (1.07-1.41) | 0.003 |
| rs11741671 | *NEURL1B* | T>C | 0.468 | 1.3 (1.07-1.59) | 0.009 | 0.095 | 0.492 | 1.22 (1.00-1.48) | 0.047 | 0.480 | 1.23 (1.07-1.41) | 0.003 |
| rs1178360 | *HDAC9* | G>C | 0.432 | 0.77 (0.62-0.96) | 0.021 | 0.168 | 0.437 | 0.83 (0.69-1.00) | 0.046 | 0.434 | 0.79 (0.69-0.91) | 0.001 |
| rs76119733 | *HDAC9* | C>CA | 0.375 | 1.31 (1.06-1.61) | 0.011 | 0.093 | 0.337 | 1.24 (1.02-1.50) | 0.030 | 0.356 | 1.27 (1.11-1.46) | 0.001 |
| rs2731546 | *HDAC9* | C>A | 0.412 | 1.33 (1.08-1.64) | 0.008 | 0.073 | 0.396 | 1.25 (1.04-1.50) | 0.017 | 0.404 | 1.30 (1.13-1.49) | <0.001 |
| rs877250 | *HDAC9* | G>A | 0.416 | 0.76 (0.61-0.96) | 0.019 | 0.182 | 0.440 | 0.8 (0.66-0.96) | 0.017 | 0.428 | 0.76 (0.66-0.88) | <0.001 |
| rs6969726 | *HDAC9* | C>G | 0.441 | 0.78 (0.62-0.97) | 0.029 | 0.199 | 0.457 | 0.83 (0.69-0.99) | 0.044 | 0.449 | 0.79 (0.68-0.91) | 0.001 |
| rs9638750 | *HDAC9* | G>A | 0.441 | 0.78 (0.62-0.97) | 0.029 | 0.199 | 0.457 | 0.83 (0.69-0.99) | 0.044 | 0.449 | 0.79 (0.68-0.91) | 0.001 |
| rs4768319 | *CNTN1* | C>A | 0.499 | 1.36 (1.11-1.67) | 0.003 | 0.035 | 0.483 | 1.26 (1.04-1.52) | 0.016 | 0.491 | 1.31 (1.14-1.50) | <0.001 |
| rs7305101 | *CNTN1* | A>G | 0.487 | 1.4 (1.14-1.71) | 0.001 | 0.012 | 0.483 | 1.23 (1.02-1.49) | 0.031 | 0.485 | 1.30 (1.14-1.50) | <0.001 |
| rs10506179 | *CNTN1* | T>C | 0.489 | 1.39 (1.13-1.70) | 0.002 | 0.015 | 0.482 | 1.22 (1.01-1.48) | 0.039 | 0.485 | 1.30 (1.13-1.49) | <0.001 |
| rs10784949 | *CNTN1* | G>A | 0.489 | 1.39 (1.13-1.70) | 0.002 | 0.015 | 0.482 | 1.22 (1.01-1.48) | 0.039 | 0.485 | 1.30 (1.13-1.49) | <0.001 |
| rs10879362 | *CNTN1* | A>T | 0.486 | 1.39 (1.14-1.71) | 0.001 | 0.021 | 0.479 | 1.22 (1.01-1.47) | 0.042 | 0.483 | 1.30 (1.13-1.49) | <0.001 |
| rs1372541 | *CNTN1* | A>G | 0.486 | 1.39 (1.13-1.71) | 0.002 | 0.021 | 0.479 | 1.21 (1.00-1.46) | 0.047 | 0.483 | 1.29 (1.13-1.48) | <0.001 |
| rs1372539 | *CNTN1* | A>T | 0.486 | 1.39 (1.13-1.71) | 0.002 | 0.021 | 0.479 | 1.21 (1.00-1.46) | 0.047 | 0.483 | 1.29 (1.13-1.48) | <0.001 |
| rs1442193 | *CNTN1* | C>T | 0.492 | 1.39 (1.13-1.70) | 0.002 | 0.015 | 0.483 | 1.25 (1.03-1.5) | 0.022 | 0.487 | 1.31 (1.14-1.50) | <0.001 |
| rs6582087 | *CNTN1* | G>A | 0.494 | 1.42 (1.16-1.74) | 0.001 | 0.009 | 0.483 | 1.25 (1.03-1.5) | 0.022 | 0.489 | 1.32 (1.15-1.51) | <0.001 |
| rs1056019 | *CNTN1* | T>C | 0.499 | 1.43 (1.17-1.76) | 0.001 | 0.010 | 0.484 | 1.27 (1.05-1.53) | 0.015 | 0.491 | 1.34 (1.17-1.54) | <0.001 |
| rs7303364 | *CNTN1* | A>G | 0.471 | 0.75 (0.61-0.91) | 0.004 | 0.035 | 0.478 | 0.81 (0.67-0.98) | 0.028 | 0.475 | 0.78 (0.68-0.89) | <0.001 |
| rs7979570 | *CNTN1* | A>C | 0.470 | 0.75 (0.61-0.92) | 0.005 | 0.056 | 0.476 | 0.82 (0.68-0.99) | 0.042 | 0.473 | 0.79 (0.69-0.90) | 0.001 |
| rs4101070 | *CNTN1* | G>T | 0.472 | 0.75 (0.62-0.92) | 0.006 | 0.056 | 0.484 | 0.82 (0.67-0.99) | 0.041 | 0.478 | 0.79 (0.69-0.90) | 0.001 |
| rs199563766 | *CNTN1* | C>CA | 0.341 | 0.75 (0.60-0.95) | 0.015 | 0.155 | 0.350 | 0.81 (0.67-0.98) | 0.028 | 0.345 | 0.78 (0.67-0.90) | 0.001 |
| rs939953001 | *CNTN1* | A>ATTTTTTTTT | 0.486 | 0.67 (0.55-0.82) | <0.001 | 0.002 | 0.493 | 0.79 (0.65-0.96) | 0.020 | 0.490 | 0.73 (0.63-0.84) | <0.001 |
| rs2006861 | *CNTN1* | A>T | 0.490 | 0.67 (0.55-0.83) | <0.001 | 0.004 | 0.489 | 0.77 (0.63-0.94) | 0.009 | 0.489 | 0.72 (0.63-0.83) | <0.001 |
| rs1346347416 | *CNTN1* | GA>G | 0.471 | 0.72 (0.59-0.88) | 0.001 | 0.015 | 0.464 | 0.76 (0.62-0.93) | 0.008 | 0.468 | 0.74 (0.65-0.85) | <0.001 |
| rs1434687 | *CNTN1* | G>A | 0.491 | 0.68 (0.56-0.84) | <0.001 | 0.005 | 0.489 | 0.77 (0.63-0.94) | 0.009 | 0.490 | 0.72 (0.63-0.83) | <0.001 |
| rs1797981 | *CNTN1* | G>T | 0.491 | 0.68 (0.56-0.84) | <0.001 | 0.005 | 0.489 | 0.77 (0.63-0.94) | 0.009 | 0.490 | 0.72 (0.63-0.83) | <0.001 |
| rs2571251 | *CNTN1* | A>G | 0.491 | 0.68 (0.56-0.84) | <0.001 | 0.005 | 0.489 | 0.77 (0.63-0.94) | 0.009 | 0.490 | 0.72 (0.63-0.83) | <0.001 |
| rs10879577 | *CNTN1* | T>C | 0.491 | 0.68 (0.56-0.84) | <0.001 | 0.005 | 0.489 | 0.77 (0.63-0.94) | 0.009 | 0.490 | 0.72 (0.63-0.83) | <0.001 |
| rs280374 | *CNTN1* | G>A | 0.491 | 0.68 (0.56-0.84) | <0.001 | 0.005 | 0.489 | 0.77 (0.63-0.94) | 0.009 | 0.490 | 0.72 (0.63-0.83) | <0.001 |
| rs11179605 | *CNTN1* | G>A | 0.491 | 0.68 (0.56-0.84) | <0.001 | 0.005 | 0.489 | 0.77 (0.63-0.94) | 0.009 | 0.490 | 0.72 (0.63-0.83) | <0.001 |
| rs800760 | *CNTN1* | T>C | 0.491 | 0.68 (0.56-0.84) | <0.001 | 0.005 | 0.489 | 0.77 (0.63-0.94) | 0.009 | 0.490 | 0.72 (0.63-0.83) | <0.001 |
| rs200389933 | *CNTN1* | T>A | 0.463 | 0.71 (0.58-0.86) | 0.001 | 0.006 | 0.456 | 0.79 (0.64-0.96) | 0.017 | 0.460 | 0.75 (0.65-0.86) | <0.001 |
| rs377240474 | *CNTN1* | A>T | 0.463 | 0.71 (0.58-0.86) | 0.001 | 0.006 | 0.456 | 0.79 (0.64-0.96) | 0.017 | 0.460 | 0.75 (0.65-0.86) | <0.001 |
| rs372864396 | *CNTN1* | T>A | 0.463 | 0.71 (0.58-0.86) | 0.001 | 0.006 | 0.456 | 0.79 (0.64-0.96) | 0.017 | 0.460 | 0.75 (0.65-0.86) | <0.001 |
| rs691564 | *CNTN1* | T>C | 0.491 | 0.68 (0.56-0.84) | <0.001 | 0.005 | 0.487 | 0.76 (0.63-0.93) | 0.008 | 0.489 | 0.72 (0.63-0.83) | <0.001 |
| rs691608 | *CNTN1* | A>G | 0.491 | 0.68 (0.56-0.84) | <0.001 | 0.005 | 0.487 | 0.76 (0.63-0.93) | 0.008 | 0.489 | 0.72 (0.63-0.83) | <0.001 |
| rs182201 | *CNTN1* | A>G | 0.463 | 0.69 (0.56-0.85) | <0.001 | 0.007 | 0.471 | 0.77 (0.63-0.94) | 0.011 | 0.467 | 0.73 (0.64-0.84) | <0.001 |
| rs444927 | *CNTN1* | G>A | 0.447 | 0.70 (0.57-0.86) | 0.001 | 0.009 | 0.446 | 0.79 (0.65-0.97) | 0.025 | 0.446 | 0.75 (0.65-0.86) | <0.001 |
| rs776885 | *CNTN1* | T>C | 0.450 | 0.7 0(0.57-0.86) | 0.001 | 0.009 | 0.449 | 0.79 (0.65-0.97) | 0.022 | 0.450 | 0.75 (0.65-0.87) | <0.001 |
| rs10408306 | *TLE2* | T>C | 0.066 | 1.73 (1.17-2.56) | 0.006 | 0.188 | 0.060 | 1.55 (1.05-2.29) | 0.027 | 0.063 | 1.59 (1.22-2.09) | 0.001 |
| rs60716314 | *TLE2* | C>T | 0.066 | 1.73 (1.17-2.56) | 0.006 | 0.188 | 0.060 | 1.55 (1.05-2.29) | 0.027 | 0.063 | 1.59 (1.22-2.09) | 0.001 |
| rs373143102 | *FCER2* | G>GTTTTT | 0.116 | 0.54 (0.38-0.79) | 0.001 | 0.089 | 0.106 | 0.67 (0.48-0.95) | 0.025 | 0.111 | 0.62 (0.48-0.80) | <0.001 |
| rs12980031 | *FCER2* | T>G | 0.125 | 0.61 (0.43-0.86) | 0.005 | 0.124 | 0.117 | 0.69 (0.50-0.95) | 0.023 | 0.121 | 0.67 (0.53-0.84) | 0.001 |
| rs889182 | *FCER2* | A>G | 0.139 | 0.65 (0.48-0.88) | 0.006 | 0.099 | 0.129 | 0.68 (0.50-0.93) | 0.015 | 0.134 | 0.68 (0.55-0.85) | 0.001 |
| rs780562176 | *FCER2* | C>CTTTA | 0.139 | 0.65 (0.48-0.88) | 0.006 | 0.099 | 0.129 | 0.68 (0.50-0.93) | 0.015 | 0.134 | 0.68 (0.55-0.85) | 0.001 |
| rs1990975 | *FCER2* | C>T | 0.139 | 0.65 (0.48-0.88) | 0.006 | 0.099 | 0.129 | 0.68 (0.50-0.93) | 0.015 | 0.134 | 0.68 (0.55-0.85) | 0.001 |

^a^ reference allele>effect allele;

^b^ The adjusted variables for Cox proportional hazards regression analysis were age, sex, smoking, drinking, AFP, Cirrhosis, Cancer embolus, BCLC stage.

Abbreviations: SNPs: single nucleotide polymorphisms; OS, overall survival; HBV: Hepatitis B Virus; HCC, hepatocellular carcinoma; MAF, minor allele frequency; HR: hazards ratio; CI: confidence interval; FPRP: false positive report rate.

**Supplementary table 3:**

Functional annotation of the 53 SNPs

| SNPs | Gene | SNPinfo^a^ | RegDB^b^ | Heploreg V4.1^c^ | | | |  |
| --- | --- | --- | --- | --- | --- | --- | --- | --- |
|  |  | function | Rank | Promoter histonemarks | Enhancer histonemarks | DNase | Motifs changed | Location |
| rs1394174 | *CNTN6* |  | 5 |  |  |  | ZID | intronic |
| rs10035202 | *NEURL1B* |  | 4 |  |  | ADRL | 6 altered motifs | missense |
| rs4868192 | *NEURL1B* | TFBS | 4 |  | 6 tissues |  | Pax-4 | intronic |
| rs35254338 | *NEURL1B* |  | 5 | BRN | 11 tissues | MUS | AIRE,Evi-1,GATA | intronic |
| rs4867682 | *NEURL1B* |  | 5 |  | 8 tissues | HRT,MUS,OVRY | PLAG1,ZID | intronic |
| rs11749132 | *NEURL1B* |  | 3a |  | HRT, GI |  | 12 altered motifs | intronic |
| rs11745123 | *NEURL1B* |  | 5 |  | GI, HRT, MUS |  | 6 altered motifs | intronic |
| rs11741671 | *NEURL1B* |  | 5 |  | GI, HRT, MUS |  | Nkx1-1,Pbx-1 | intronic |
| rs1178360 | *HDAC9* |  | 7 |  |  |  | HMG-IY,NF-AT,RBP-Jkappa | intronic |
| rs76119733 | *HDAC9* |  | 6 |  | BLD |  | 5 altered motifs | intronic |
| rs2731546 | *HDAC9* |  | 5 |  |  | SKIN | 8 altered motifs | intronic |
| rs877250 | *HDAC9* |  | 5 | FAT | FAT | KID | LBP-9 | intronic |
| rs6969726 | *HDAC9* |  | 7 |  |  |  |  | intronic |
| rs9638750 | *HDAC9* |  | 5 |  |  |  | YY1 | intronic |
| rs4768319 | *CNTN1* |  | 6 |  |  |  |  | intronic |
| rs7305101 | *CNTN1* |  | 6 |  |  |  | 9 altered motifs | intronic |
| rs10506179 | *CNTN1* |  | 5 |  |  |  | 6 altered motifs | intronic |
| rs10784949 | *CNTN1* |  | 5 |  |  | IPSC | 6 altered motifs | intronic |
| rs10879362 | *CNTN1* |  | 3a |  |  |  | 22 altered motifs | intronic |
| rs1372541 | *CNTN1* |  | 6 |  |  |  | Irf,Pou5f1,STAT | intronic |
| rs1372539 | *CNTN1* |  | 7 |  |  |  | Evi-1 | intronic |
| rs1442193 | *CNTN1* |  | 6 |  |  |  | 15 altered motifs | intronic |
| rs6582087 | *CNTN1* |  | 7 |  |  |  | GATA | intronic |
| rs1056019 | *CNTN1* |  | 7 |  |  |  | 5 altered motifs | synonymous |
| rs7303364 | *CNTN1* |  | 5 |  |  |  | 33 altered motifs | intronic |
| rs7979570 | *CNTN1* |  | 7 |  |  |  | NF-AT | intronic |
| rs4101070 | *CNTN1* |  | 5 |  |  |  | 8 altered motifs | intronic |
| rs199563766 | *CNTN1* |  | 5 |  | SKIN, PLCNT | SKIN | 13 altered motifs | intronic |
| rs939953001 | *CNTN1* |  | 6 |  |  |  |  |  |
| rs2006861 | *CNTN1* |  | 3b |  |  | LIV | 7 altered motifs | intronic |
| rs1346347416 | *CNTN1* |  | 5 |  | SKIN, PLCNT | SKIN | 13 altered motifs | intronic |
| rs1434687 | *CNTN1* |  | 3a |  |  |  | Mef2 | intronic |
| rs1797981 | *CNTN1* |  | 7 |  |  |  |  | intronic |
| rs2571251 | *CNTN1* |  | 7 |  |  |  |  | intronic |
| rs10879577 | *CNTN1* |  | 6 |  |  |  | 5 altered motifs | intronic |
| rs280374 | *CNTN1* |  | 4 |  | STRM, GI | MUS | Barhl1,GR | intronic |
| rs11179605 | *CNTN1* |  | 6 |  |  |  | 4 altered motifs | intronic |
| rs800760 | *CNTN1* |  | 4 |  | STRM |  |  | intronic |
| rs200389933 | *CNTN1* |  | 6 |  |  |  | 10 altered motifs | intronic |
| rs377240474 | *CNTN1* |  | 6 |  |  |  |  |  |
| rs372864396 | *CNTN1* |  | 6 |  |  |  |  |  |
| rs691564 | *CNTN1* |  | 7 |  |  | LNG | EBF,Nkx2 | intronic |
| rs691608 | *CNTN1* |  | 7 |  |  |  | Pou3f2 | intronic |
| rs182201 | *CNTN1* |  | 6 |  |  |  |  | intronic |
| rs444927 | *CNTN1* | miRNA | 7 |  |  |  | Ik-1,NF-kappaB,Znf143 | 3'-UTR |
| rs776885 | *CNTN1* |  | 7 |  |  |  | CTCF,Hsf,p300 |  |
| rs10408306 | *TLE2* |  | 5 |  |  | IPSC,BLD | BDP1,Irf,p300 | intronic |
| rs60716314 | *TLE2* |  | 5 |  | BLD | IPSC,BLD | BDP1,Irf,Nr2f2 | intronic |
| rs373143102 | *FCER2* |  | 2b |  |  |  |  | Intron Variant |
| rs12980031 | *FCER2* |  | 2b | BLD | 4 tissues | 20 tissues | 5 altered motifs | intronic |
| rs889182 | *FCER2* |  | 1f | BLD |  | BLD,BLD | NRSF | intronic |
| rs780562176 | *FCER2* |  |  |  |  |  |  |  |
| rs1990975 | *FCER2* | TFBS | 4 | BLD | IPSC, BLD, ADRL | BLD,BLD,BLD |  | ntronic |

^a^ http://snpinfo.niehs.nih.gov/snpinfo/snpfunc.htm;

^b^ http://www.regulomedb.org/index;

^c^  http://archive.broadinstitute.org/mammals/haploreg/haploreg.php
